# Natural Killer Cells from Patients with Recombinase-Activating Gene and Non-Homologous End Joining Gene Defects Comprise a Higher Frequency of CD56^bright^ NKG2A^+++^ Cells, and Yet Display Increased Degranulation and Higher Perforin Content

**DOI:** 10.3389/fimmu.2017.00798

**Published:** 2017-07-17

**Authors:** Kerry Dobbs, Giovanna Tabellini, Enrica Calzoni, Ornella Patrizi, Paula Martinez, Silvia Clara Giliani, Daniele Moratto, Waleed Al-Herz, Caterina Cancrini, Morton Cowan, Jacob Bleesing, Claire Booth, David Buchbinder, Siobhan O. Burns, Talal A. Chatila, Janet Chou, Vanessa Daza-Cajigal, Lisa M. Ott de Bruin, Maite Teresa de la Morena, Gigliola Di Matteo, Andrea Finocchi, Raif Geha, Rakesh K. Goyal, Anthony Hayward, Steven Holland, Chiung-Hui Huang, Maria G. Kanariou, Alejandra King, Blanka Kaplan, Anastasiya Kleva, Taco W. Kuijpers, Bee Wah Lee, Vassilios Lougaris, Michel Massaad, Isabelle Meyts, Megan Morsheimer, Benedicte Neven, Sung-Yun Pai, Nima Parvaneh, Alessandro Plebani, Susan Prockop, Ismail Reisli, Jian Yi Soh, Raz Somech, Troy R. Torgerson, Yae-Jaen Kim, Jolan E. Walter, Andrew R. Gennery, Sevgi Keles, John P. Manis, Emanuela Marcenaro, Alessandro Moretta, Silvia Parolini, Luigi D. Notarangelo

**Affiliations:** ^1^Laboratory of Host Defenses, Division of Intramural Research, National Institute of Allergy and Infectious Diseases, National Institutes of Health, Bethesda, MD, United States; ^2^Department of Molecular and Translational Medicine, University of Brescia, Brescia, Italy; ^3^“A. Nocivelli Institute for Molecular Medicine”, Pediatric Clinic, University of Brescia, Azienda Socio Sanitaria Territoriale degli Spedali Civili di Brescia, Brescia, Italy; ^4^Hospital de Niños Ricardo Gutiérrez, Buenos Aires, Argentina; ^5^Department of Pediatrics, Faculty of Medicine, Kuwait University, Kuwait City, Kuwait; ^6^DPUO, Division of Immuno-Infectivology, University Department of Pediatrics, Bambino Gesù Children’s Hospital, Rome, Italy; ^7^School of Medicine, University of Tor Vergata, Rome, Italy; ^8^Pediatric Allergy Immunology and Blood and Marrow Transplant Division, University of California, San Francisco, Benioff Children’s Hospital, San Francisco, CA, United States; ^9^Division of Hematology/Oncology, Cincinnati Children’s Hospital Medical Center, Cincinnati, OH, United States; ^10^Institute for Immunity and Transplantation, University College London, London, United Kingdom; ^11^Division of Pediatric Hematology, Children’s Hospital Orange County, University of California Irvine, Orange County, CA, United States; ^12^Department of Immunology, Royal Free London NHS Foundation Trust, London, United Kingdom; ^13^Division of Immunology, Boston Children’s Hospital, Boston, MA, United States; ^14^Division of Allergy and Immunology, Southwestern Medical Center, University of Texas, Dallas, TX, United States; ^15^Division of Hematology/Oncology/BMT, Children’s Mercy Hospital & Clinics, Kansas City, MO, United States; ^16^Department of Pediatrics, Brown University, Providence, RI, United States; ^17^Laboratory of Clinical Infectious Diseases, National Institute of Allergy and Infectious Diseases, National Institutes of Health, Bethesda, MD, United States; ^18^Department of Paediatrics, National University Hospital, Singapore, Singapore; ^19^Department of Immunology-Histocompatibility, “Aghia Sophia” Children’s Hospital, Athens, Greece; ^20^Division of Pediatric Immunology, Hospital Luis Calvo Mackenna, Santiago, Chile; ^21^Department of Pediatrics, Division of Allergy and Immunology, Hofstra Northwell School of Medicine, Hofstra University, Great Neck, NY, United States; ^22^Department of Pediatric Hematology, Immunology and Infectious Diseases, Emma Children’s Hospital, Academic Medical Center (AMC), University of Amsterdam, Amsterdam, Netherlands; ^23^Department of Experimental and Clinical Sciences, University of Brescia, Brescia, Italy; ^24^Department of Pediatrics, University Hospitals Leuven, Leuven, Belgium; ^25^Transplantation Branch, Division of Allergy, Immunology and Transplantation, National Institute of Allergy and Infectious Diseases, National Institutes of Health, Rockville, MD, United States; ^26^Pediatric Hematology-Immunology Department, Hospital Necker-Enfants Malades, Institute Imagine, AP-HP, Paris Descartes University, Sorbonne-Paris-Cité, Paris, France; ^27^Division of Hematology-Oncology, Boston Children’s Hospital, Boston, MA, United States; ^28^Division of Allergy and Clinical Immunology, Department of Pediatrics, Tehran University of Medical Sciences, Tehran, Iran; ^29^Research Center for Immunodeficiencies, Children’s Medical Center, Tehran, Iran; ^30^Bone Marrow Transplant Service, Department of Pediatrics, Memorial Sloan Kettering Cancer Center, New York, NY, United States; ^31^Division of Pediatric Immunology and Allergy, Meram Medical Faculty, Necmettin Erbakan University, Konya, Turkey; ^32^Pediatric Immunology Unit, The Edmond and Lily Safra Children’s Hospital, Sheba Medical Center, Tel Hashomer, Sackler Faculty of Medicine, Tel Aviv University, Tel Aviv, Israel; ^33^Department of Pediatrics and Immunology, Seattle Children’s Hospital, University of Washington, Seattle, WA, United States; ^34^Division of Infectious Diseases and Immunodeficiency, Department of Pediatrics, Samsung Medical Center, School of Medicine, Sungkyunkwan University, Seoul, South Korea; ^35^Division of Pediatric Allergy/Immunology, University of South Florida at Johns Hopkins All Children’s Hospital, St. Petersburg, FL, United States; ^36^Department of Paediatric Immunology, Great North Children’s Hospital, Newcastle Upon Tyne, United Kingdom; ^37^Institute of Cellular Medicine, Newcastle University, Newcastle Upon Tyne, United Kingdom; ^38^Department of Laboratory Medicine, Boston Children’s Hospital, Boston, MA, United States; ^39^Molecular Immunology Laboratories, Department of Experimental Medicine, University of Genoa, Genoa, Italy

**Keywords:** natural killer cells, recombinase-activating genes, non-homologous end joining, immunodeficiency, CD56, interferon-γ, degranulation

## Abstract

Mutations of the recombinase-activating genes 1 and 2 (*RAG1* and *RAG2*) in humans are associated with a broad range of phenotypes. For patients with severe clinical presentation, hematopoietic stem cell transplantation (HSCT) represents the only curative treatment; however, high rates of graft failure and incomplete immune reconstitution have been observed, especially after unconditioned haploidentical transplantation. Studies in mice have shown that *Rag^−/−^* natural killer (NK) cells have a mature phenotype, reduced fitness, and increased cytotoxicity. We aimed to analyze NK cell phenotype and function in patients with mutations in *RAG* and in non-homologous end joining (NHEJ) genes. Here, we provide evidence that NK cells from these patients have an immature phenotype, with significant expansion of CD56^bright^ CD16^−/int^ CD57^−^ cells, yet increased degranulation and high perforin content. Correlation was observed between *in vitro* recombinase activity of the mutant proteins, NK cell abnormalities, and *in vivo* clinical phenotype. Addition of serotherapy in the conditioning regimen, with the aim of depleting the autologous NK cell compartment, may be important to facilitate engraftment and immune reconstitution in patients with RAG and NHEJ defects treated by HSCT.

## Introduction

Natural killer (NK) cells are effector and immunomodulatory cells of the innate immune system. Through recognition of HLA class I molecules *via* activating and inhibitory killer cell immunoglobulin-like receptors (KIRs), and upon release of cytolytic granules, NK cells mediate killing of tumor and virus-infected cells and editing of dendritic cells, and play an important role in graft-versus-leukemia and graft rejection after hematopoietic stem cell transplantation (HSCT) (1–4). Furthermore, they can modulate immune responses by secreting chemokines and cytokines (5, 6).

Human peripheral blood contains two major NK cell subsets that can be distinguished based on the density of CD56 and CD16 expression on the cell surface: CD56^bright^ CD16^−/low^ and CD56^dim^ CD16^bright^ cells. These two NK cell subsets differ for the expression pattern of various other cell surface and intracellular molecules (7). In particular, CD56^bright^ cells express NKp46, CD94/NKG2A, and CCR7 at higher levels than CD56^dim^ NK cells, whereas CXCR1 and KIRs are more abundantly expressed by CD56^dim^ cells. Furthermore, CD56^bright^ and CD56^dim^ NK cells have distinct functional properties, with CD56^bright^ cells being potent producers of cytokines, and CD56^dim^ cells being active mediators of natural and antibody-dependent cellular cytotoxicity, as also reflected by higher intracellular levels of perforin and granzymes (8, 9). In healthy adults, CD56^bright^ cells comprise a minority (5–10%) of all circulating NK cells, but because they express CCR7, they are attracted to secondary lymphoid organs where they represent the predominant NK subset (10, 11). A subset of CD56^low^ KIR^+^ NK cells, expressing CD57 represent terminally differentiated NK cells, whereas a further subset expressing the CD56^−^ CD16^+^ CD57^+^ KIR^+^ phenotype are thought to represent exhausted NK cells (12).

*RAG1* and *RAG2* mutations in humans are associated with a broad spectrum of clinical and immunological phenotypes, including T^−^ B^−^ severe combined immune deficiency (SCID) (13), Omenn syndrome (OS) (14), atypical SCID (AS) (15–17), and combined immune deficiency with granuloma and/or autoimmunity (CID-G/A) (18–21). We have previously shown that the severity of the clinical and immunological phenotype in patients with *RAG* mutations correlates with the residual recombination activity of the mutant recombinase-activating gene (RAG) protein (22), which may differently affect diversity and composition of T and B cell receptor repertoires (23), whereas NK cell differentiation proceeds unaffected. For patients with severe *RAG* mutations presenting with SCID, OS, or AS, HSCT represents the only option of definitive cure; however, an increased rate of allograft rejection has been observed as compared to patients with other forms of SCID (24, 25). An important role of NK lymphocytes in mediating rejection of bone marrow allografts has been known for decades (26), but why patients with RAG deficiency would have a higher risk of graft rejection than other forms of NK^+^ SCID (such as IL7R or CD3 deficiency) remains unknown.

Although *RAG* genes are not required for NK cell development, data in mice indicate that Rag deficiency affects NK cell phenotype and function. It has been shown that expression of the *RAG* genes begins in common lymphoid progenitor cells that give rise to T, B, and NK cells (27–29). Studies in mice harboring transgenic reporters for Rag expression or recombinase activity have demonstrated the existence of two populations of mature NK cells: those that have been exposed to transient Rag expression during lymphoid differentiation (here termed as Rag^past^) and NK cells that were not previously exposed to Rag (Rag^naive^ NK cells) (30). These two populations differ for their proliferative capacity and interleukin-2 (IL-2)-mediated Stat5 phosphorylation, and a progressive decrease in the proportion of Rag^past^ cells has been observed during NK cell differentiation (29). Furthermore, Rag^naive^ NK cells display an activated phenotype, increased cytotoxicity, and enhanced apoptosis, thereby resulting in poor survival and impaired DNA damage response as compared to their Rag^past^ counterpart (30). It has been postulated that Rag expression in lymphoid progenitors would favor selection of cells with optimal levels of expression of proteins involved in DNA break repair, including ARTEMIS and DNA ligase 4 (LIG4), thereby marking functionally distinct subsets of NK cells, and providing Rag^past^ NK cells with improved survival and “fitness.” If confirmed also in humans, the hypothesis that RAG deficiency results in the presence of NK cells with a distinctive phenotype and enhanced cytotoxic potential, would provide novel mechanistic insights to account for the high rate of primary graft failure, incomplete T cell reconstitution, and lack of B cell engraftment that are frequently observed following haploidentical HSCT for RAG deficiency, unless chemotherapy is used (25). To test this hypothesis, we have analyzed NK cell phenotype and function in a large cohort of patients with mutations in *RAG* and in other genes involved in non-homologous end joining (NHEJ), presenting with a variable severity of clinical and immunological manifestations, as compared to healthy donors of comparable age, and to patients with other forms of T or B cell lymphopenia.

## Materials and Methods

### Patients and Controls

The patient population consisted of 66 subjects with molecularly confirmed biallelic mutations in gene involved in V(D)J recombination, in particular 35 patients with *RAG1*, 11 with *RAG2*, 15 with *DCLRE1C* (ARTEMIS), 3 with *LIG4*, and 2 with *NHEJ1* (Cernunnos/XLF) mutations. Based on the clinical and immunological phenotype (Table 1), these patients were assigned to the following subgroups: T^−^ B^−^ NK^+^ SCID (*n* = 19), OS (*n* = 11), AS (*n* = 13), and delayed onset combined immune deficiency (CID, *n* = 23). For the purposes of analysis, patients with OS and with AS, were combined into a single group (OS/AS, *n* = 24).

**Table 1:** Clinical, molecular, and immunological features of patients with mutations in *RAG* or non-homologous end-joining (NHEJ) genes.

Patient ID	Phenotype	Age	Gene defect and mutation	Lymphocyte subsets (cells/μL)					IgG (mg/dL)	IgA (mg/dL)	IgM (mg/dL)	IgE (kU/L)	Proliferation to phytohemagglutinin	Cytomegalovirus (CMV) status	EpsteinBarr virus (EBV) status	Infections	Autoimmunity	Granulomas	Skin rash
				CD3	CD4	CD8	CD19	CD16/56											
P1	Severe combined immune deficiency (SCID)	9 months	RAG1: p.R332X; p.R561H	185	74	24	0	166	883^^^	<7	<5	<2	Absent	neg	neg	Rotavirus, *Pseudomonas*, Enterococcus enteritis	No	No	No
P2	SCID	2 months	RAG2: p.I218N; p.I218N	25	23	0	1	155	<7	<7	<5	nd	nd	neg	neg	PJP	No	No	No
P3	SCID	10 months	RAG2: p.T125Rfs*10; p.T125Rfs*10	230	230	4	0	352	929^^^	<7	<5	<1	Absent	neg	neg	PJP, metapneumovirus, oral candidiasis	No	No	No
P4	SCID	2 months	RAG1: p.R841Q; p.F974L	34	30	4	265	395	371	22	67	38	Markedly reduced	neg	neg	No	No	No	No
P5	SCID	2 weeks	RAG1: p.R507W; p.S966T	286	111	190	16	1,027	235	<15	18	nd	Reduced	pos	nd	CMV viremia	No	No	No
P6	SCID	6 months	RAG1: p.E965X; p.E965X	2	2	0	0	252	<7	<7	<5	nd	Absent	neg	neg	*Klebsiella pneumonia*	No	No	No
P7	SCID	2 months	RAG1: p.M605I/R561C; p.F972S	2	1	0	0	275	325	<7	<5	nd	nd	pos	neg	RSV bronchiolitis, adenovirus pneumonia, CMV	No	No	No
P8	SCID	6 months	RAG2: p.C41W; p.C41W	5	1	0	1	804	71	<7	<5	<1	absent	neg	neg	Disseminated BCG	No	No	No
P9	SCID	5 months	RAG1: p.G720D; p.G720D	9	9	0	0	449	766	<7	<5	<1	absent	neg	neg	Cutaneous *S. aureus* abscess	No	No	No
P10	SCID	5 months	RAG1: p.C470Lfs*55; p.C470Lfs*55	236	90	146	0	80	161	<7	<5	nd	nd	neg	neg	No	No	No	No
P11	SCID	1 week	RAG1: p.C176F; p.C176F	23	13	1	573	1,449	754	<7	<5	7.8	Absent	neg	neg	No	No	No	No
P12	SCID	14 months	DCLRE1C: exon1_3del; exon1_3del	11	0	4	10	1,248	722^^^	29	16	<1	Absent	neg	neg	Disseminated BCG	No	AFB ^+^ necrotizing granulomatous lymphadenitis	No
P13	SCID	3 months	DCLRE1C: exon1_3del; exon1_3del	1,076	1,070	10	0	904	50	<7	<5	nd	nd	neg	neg	RSV pneumonia	No	No	Yes
P14	SCID	5 months	DCLRE1C: p.K157Kfs*13; p.K157Kfs*13	215	152	61	40	287	540	38	106	nd	Markedly reduced	neg	neg	*Pseudomonas* bacteremia, recurrent otitis, skin abscesses, oral candidiasis	No	No	Papular rash
P15	SCID	1 week	DCLRE1C: exon1_3del; exon1_3del	0	0	0	10	840	nd	nd	nd	nd	nd	nd	nd	Sepsis	No	No	No
P16	SCID	23 months	DCLRE1C: p.Y199X; p.Y199X	107	50	63	0	498	1,350^^^	15	<5	<1	Absent	neg	neg	PJP, RSV pneumonia, cutaneous *Pseudomonas* infection, candidiasis	No	No	No
P17	SCID	28 months	DCLRE1C: p.Y199X; p.Y199X	46	39	7	0	291	1,060	620	nd	nd	Absent	neg	neg	No	No	No	np
P18	SCID	4 months	DCLRE1C: p.S32C; IVS11, +5g>a	7	1	0	1	598	115	<7	nd	nd	Absent	neg	neg	Adenovirus hepatitis, oral ulcers	No	No	No
P19	SCID	10 months	DCLRE1C: p.F393fs*8; p.F393fs*8	13,997 (maternal)	9,851	3,221	29	274	830^^^	<7	nd	nd	nd	pos	nd	Oral candidiasis	No	No	Generalized erythroderma
P20	Omenn syndrome (OS)	2 months	RAG1: p.R396H; p.R396H	15,760	6,390	9,465	0	1,958	300	<7	<5	1,744	Reduced	pos	neg	Candidiasis, *E. coli* UTI	No	No	Generalized erythroderma
P21	OS	1 month	RAG1: p.W959X; p.W959X	3,424	3,368	0	0	5,003	390	22	16	<2	Absent	neg	neg	Rhinovirus	No	No	Generalized erythroderma
P22	OS	2 months	RAG1: p.R410Q; p.M435V	19,230	10,286	8,215	0	816	1,120	<7	101	>5,000	Markedly reduced	neg	neg	Rhinovirus	No	No	Generalized erythroderma
P23	OS	2 months	RAG1: p.R561C; p.R716W	4,667	3,976	696	0	7,231	<7	<7	<5	233	Absent	neg	neg	No	No	No	Generalized erythroderma
P24	OS	4 months	RAG1: p.R396C; p.R404Q	15,751	8,850	6,957	0	1,389	258	216	<5	2,181	Absent	neg	neg	PJP, rhinovirus	No	No	Generalized erythroderma
P25	OS	2 months	RAG1: p.R561H; p.R624H	11,140	6,204	4,931	0	4,564	100	6	302	>5,000	Markedly reduced	pos	neg	No	Anti-smooth muscle ab	No	Generalized erythroderma
P26	OS	6 months	RAG2: p.G95V; p.E480X	2,671	2,351	855	0	36	102	116	235	<5	Markedly reduced	neg	neg	Impetigo, otitis	No	No	Generalized erythroderma
P27	OS	1 month	RAG1: p.R142X; p.M458fs*33	5,105	5,029	228	15	4,458	634*	17	8	<2	Markedly reduced	pos	neg	Recurrent URTI/LRTI	No	No	Generalized erythroderma
P28	OS	1 month	RAG1: p.S259Afs*5; p.S259Afs*5	170	155	15	0	320	815	<7	5	14.4	Markedly reduced	neg	neg	*Enterococcus faecalis* bacteremia	Intermittent neutropenia	No	Generalized erythroderma
P29	OS	4 months	RAG1: p.R332X; p.L1025fs*39	34,115	22,416	8,364	0	1,338	<7	<7	<5	600	Reduced	neg	neg	Enterovirus pneumonia, Moraxella bacteremia	No	No	Generalized erythroderma
P30	OS	4 months	DCLRE1C: exon1_3del; spling defect intron6	26,295	5,085	19,382	0	1,498	120	602	26	192	nd	neg	neg	PJP, rhinovirus	No	No	Generalized erythroderma
P31	Atypical SCID (AS)	Not known	RAG2: p.R123C; p.R123C	1,056	96	780	23	67	206	11	14	1	nd	neg	pos	Skin abscess, recurrent pneumonia, candidiasis	Lymphadenopathy	No	No
P32	AS	4 months	RAG1: p.H375D; p.H375D	870	610	210	350	390	370	nd	89	nd	Absent	nd	nd	Candidiasis	No	No	Candida rash
P33	AS	17 months	RAG1: p.R396C; p.M435V	360	180	150	31	370	1,036	<7	145	77	Markedly reduced	neg	neg	Vaccine strain varicella zoster virus (VZV), chronic diarrhea	AIHA, vasculitis	No	Vasculitis
P34	AS	3 years	RAG1: p.H612R; p.A857V	2,063	225	878	612	1,068	2,080	<7	172	1.9	Reduced	pos	neg	Adenovirus pneumonia, *Klebsiella pneumonia*, candidiasis	ANA, TPO Ab	No	No
P35	AS	2 years	RAG1: p.F939C; p.F939C	729	110	266	41	733	1,260	55	215	78	normal	nd	nd	BCG infection	No	No	No
P36	AS	2 months	RAG1: p.T173fs*27; p.T173fs*27	399	93	209	3	1,205	392	10	<5	113	absent	pos	neg	Pneumonia	AIHA	No	No
P37	AS	16 months	RAG2: p.G35A; p.G35A	716	277	102	105	209	229	75	327	nd	Reduced	neg	pos	Recurrent pneumonia (P*seudomonas, Klebsiella, H. influenzae*), nail candidiasis	AIHA, psoriasis	NO	Psoriasis
P38	AS	25 months	RAG1: p.R841W; p.R841W	950	220	20	121	665	538^^^	146	54	2	Normal	neg	neg	Adenovirus, rhinovirus	Tubulointerstitial nephritis with lymphocytic infiltrate	No	No
P39	AS	13 months	RAG1: p.K86Vfs*33; p.K86Vfs*33	1,010	80	840	620	460	2,420	194	328	5.6	Markedly reduced	pos	pos	P*seudomonas* sepsis, CMV, BCGitis	Miller-Fisher	No	No
P40	AS	4 years	DCLRE1C: p.T65I; p.T65I	558	207	243	36	108	560	19	54	5	nd	nd	nd	HPV	No	No	Warts
P41	AS	10 years	DCLRE1C: p.T65I; p.T65I	683	268	390	24	403	1,190	<7	87	5	nd	nd	nd	HPV, recurrent URTI/LRTI	No	No	Warts
P42	AS	2 years	DCLRE1C: p.T65I; p.T65I	547	217	268	22	226	240	<7	35	5	Absent	nd	nd	HPV, BCG	No	No	Warts
P43	AS	9 months	NHEJ1: p.R57X; p.R57X	426	299	98	259	265	600	<7	949	nd	Reduced	neg	neg	Adenovirus	No	No	No
P44	CID	6 years	RAG2: p.T215I; p.T215I	840	560	180	70	190	2,480^^^	<7	170	nd	Reduced	nd	pos	Recurrent URTI, intermittent diarrhea	Neutropenia	No	No
P45	CID	19 years	RAG2: p.G35V; p.M322T	457	236	184	165	5,781	<100	<7	<5	nd	Reduced	neg	pos	Single episode of RTI requiring admission at age 13 years	No	Skin granulomatosis	Skin granulomas
P46	CID	11 years	RAG1:p.R404Q; p.R404Q	4,520	3,300	1,220	61	1,004	<200	<7	30	nd	Absent	pos	pos	CMV retinitis, esophageal candidiasis, EBV	Colitis	Colon granulomatosis	No
P47	CID	8 years	RAG1: p.H612R; p.H612R	526	361	144	373	106	859^^^	<7	<5	1	Markedly reduced	na	na	Recurrent pneumonia, MAC, cellulitis	AIHA	No	No
P48	CID	17 years	RAG1: p.K86Vfs*33; p.H612R	581	530	80	460	450	390	<7	38	<1	Reduced	pos	pos	Herpes zoster, *H. influenzae, Corynebacterium propinquum*, P*seudomonas*	ITP, vitiligo	Lung granulomatosis	No
P49	CID	11 years	RAG1: p.L514R; p.L514R	2,831	1,658	837	0*	309	<140	<7	130	<18	normal	neg	pos	Recurrent URTI/LRTI, warts, molluscum, EBV-LPD	Autoimmune cytopenias	Granulomas in liver and lungs	Warts, molluscum
P50	CID	31 years	RAG2: p.F62L; p.F62L	380	225	162	78	215	1,000^^^	<7	25	nd	nd	na	na	Disseminated VZV, cryptococcus meningitis, URTI, *Pseudomonas* pneumonia	No	Lung granulomatosis	
P51	CID	12 years	RAG1: p.R624H; p.Y728H	665	406	219	23	177	8	<7	10	<2	Normal	neg	pos	Moderately severe VZV, zoster, recurrent URTI/LRTI	No	No	No
P52	CID	9 years	RAG2: p.V8I; p.D400H	2,253	1,275	774	293	205	910	257	33	29	Normal	neg	neg	*S. pneumoniae* sepsis, severe VZV infection, recurrent URTI/LRTI, perianal abscess	No	No	Severe atopic dermatitis
P53	CID	16 years	RAG1: p.H612R; p.H612R	629	390	141	69	60	637	37	45	nd	Normal	neg	neg	Recurrent otitris, recurrent pneumonia, *S. aureus* skin infection	Alopecia areata, AIHA, neutropenia	No	No
P54	CID	9 years	RAG1: p.R474C; p.L506F	890	500	400	300	130	560	20	60	5	Normal	neg	pos	VZV pneumonia, bronchiectasis	No	No	No
P55	CID	39 years	RAG1: p.R108X; p.W522C	532	336	179	14	166	673	106	69	<1	nd	nd	nd	Pneumonia, warts, orolabial HSV, oral candidiasis, MRSA skin infection	Alopecia, autoimmune hypothyroidism, autoimmune hypogonadism	Vocal cord granuloma	No
P56	CID	16 years	RAG1: p.H375D; p.Y562C	522	468	18	72	6	1,346^^^	420	320	nd	Reduced	neg	neg	Pneumonia	ITP, neutropenia	Granulomatosis of skin, liver, spleen, lungs	No
P57	CID	10 years	RAG1: p.R410W; p.H375D	273	169	42	56	150	597^^^	71	87	nd	Normal	neg	neg	Recurrent LRTI	Alopecia totalis, vitiligo, myasthenia gravis	No	No
P58	CID	40 years	RAG1: p.R108X; p.W522C	362	235	117	0*	106	976	100	33	0	nd	neg	neg	Recurrent URTI/LRTI, warts, mouth sores, oral and nail candidiasis	Alopecia, vitiligo	No	No
P59	CID	36 years	RAG2: p.N173S; p.E437K	260	204	54	0*	134	1,050^^^	17	17	0	Reduced	neg	pos	Septis arthritis, recurrent pneumonia, warts	Alopecia, myositis	No	Psoriatic rash
P60	CID	30 months	DCLRE1C: p.G126D; p.L70del	2,800	1,040	2,340	520	510	630^^^	<7	210	<2	Markedly reduced	neg	pos	Echovirus, adenovirus	AIHA	no	Perianal rash
P61	CID	2 years	DCLRE1C: p.T65I; p.T65I	540	160	240	16	1,320	135	<7	15	nd	nd	nd	nd	Recurrent URTI/LRTI, aphtous stomatitis	Lupus vulgaris	No	Lupus vulgaris
P62	CID	12 years	DCLRE1C: p.S147fs*6; p.S147fs*6	260	170	90	2	1,635	173	<7	11	2	nd	neg	neg	JC virus-associated PML	Vasculitis	Cutaneous granulomatous vasculitis	Vasculitis
P63	CID	7 years	NHEJ1: p.R57G; p.R57G	693	319	330	44	195	60	<7	104	nd	Reduced	neg	neg	Recurrent URTI/LRTI	No	No	No
P64	CID	3 years	Ligase 4 (LIG4): p.R278H; p.R278H	3,088	1,191	1,832	46	2,081	1,400^^^	<7	167	nd	Normal	neg	pos	Chronic calicivirus	No	No	No
P65	CID	8 years	LIG4: p.K424Rfs*20; p.R278H	513	214	289	96	438	622	<7	<10	2	Reduced	pos	pos	Pneumonia, otitis	No	No	No
P66	CID	17 years	LIG4: p.K449Q; p.R814X	771	308	410	12	140	149	<7	<5	<1	Reduced	neg	neg	Recurrent URTI/LRTI, bronchiectasis, skin ringworm infection	No	No	No

*^^^, on immunoglobulin replacement therapy; Ab, antibody; AIHA, autoimmune hemolytic anemia; ANA, anti-nuclear antibodies; BCG, bacillus calmette-guerin; CMV, cytomegalovirus; HPV, human papillomavirus; ITP, immune thrombocytopenia; LPD, lymphoproliferative disease; LRTI, lower respiratory tract infection; PJP, Pneumocystis jiroveci pneumonia; RSV, respiratory syncitial virus; TPO, thyroid peroxidase; URTI, upper respiratory tract infection; UTI, urinary tract infection; VZV, Varicella zoster virus*.

In addition, 22 patients with various other conditions characterized by numerical and/or functional defects of T lymphocytes served as a control for T cell deficiency. This group of other T cell defects (TCDs) included four patients with *CD3G* mutations, two patients with Di George syndrome, two patients with IL7R deficiency, and one patient each with mutations in *MSN, JAK3, IL2RG, CD3D, CD3E, RMRP* genes, or with MHC class II deficiency. In seven cases with T cell deficiency, the underlying diagnosis was unknown, including two infants with severe T cell lymphopenia diagnosed at birth with low T cell receptor excision circle (TRECs). Based on established criteria (31), 7 of these patients met definition of SCID (with <300 T cells/μL), and the remaining 15 had either less severe numerical TCDs or had functional T cell abnormalities. None of these patients with TCD carried mutations in *RAG1/2, DCLRE1C, PRKDC, LIG4*, or *NHEJ1* genes.

As a control for B cell lymphopenia, nine patients with molecularly confirmed mutation in the *BTK* gene, causing X-linked agammaglobulinemia (XLA), were also studied.

Finally, 19 healthy infants (age ≤2 years) and 29 healthy subjects of >2 years of age served as normal controls for patients with SCID and OS/AS, or for patients with CID, respectively.

The study was approved by the Institutional Review Boards of Boston Children’s Hospital, the National Institute of Allergy and Infectious Diseases (protocols 93-I-0119 and 16-I-N139), and of all other referring centers. Blood samples were obtained upon written informed consent of the subject or, in the case of minors, of their parents or legal guardians. Because of limitations in blood volume and/or in the number of cells, not all analyses could be performed in each individual included in the study.

### Analysis of NK Cell Phenotype

Peripheral blood was collected in EDTA- or heparin-containing vacutainers. Red blood cells were lysed from 200 µl of EDTA-blood using RBC lysis buffer (eBioscience). Cells were washed with FACS buffer (PBS containing 2% FBS), then equally distributed in five tubes and incubated for 1 h at 4°C with primary antibodies (Ab), as described below. After washing with FACS buffer, cells were incubated for 30 min at 4°C with secondary antibodies, washed again with FACS buffer, and incubated for 30 min at 4°C with conjugated antibodies. After washing, cells were re-suspended in FACS buffer and immediately analyzed on LSR Fortessa Flow Cytometer (BD) using FACS Diva v.6.1.3 software (BD). NK cells were defined as CD56^+^ CD3^−^ CD20^−^ CD14^−^ cells. Final analysis on the expression of NK cell markers was performed using FloJo v.10.2 (TreeStar). The antibodies used to define NK cell phenotype were either commercially available or were generated in the laboratories of Alessandro Moretta and Silvia Parolini.

To analyze the expression of NK cell surface markers, five different tubes were prepared (Table 2), each of which contained FITC-conjugated antibodies directed against CD3, CD20, and CD14, to gate out T cells, B cells, and monocytes. Furthermore, tube #1 contained isotype controls, whereas tubes #2–5 contained antibodies to NK cell markers.

**Table 2:** Combination of antibodies used to characterize natural killer cell phenotype.

Tube #	Antibody	Source
1	IgG1 isotype control	Biolegend
	IgG2a isotype control	Biolegend
	IgG2b isotype control	Biolegend
	PE-conjugated goat anti-mouse IgG1	Southern Biotech
	PE-conjugated goat anti-mouse IgG2a	Southern Biotech
	PE/Cy7-conjugated goat anti-mouse IgG2b	Southern Biotech
	FITC-conjugated anti-CD3	Beckman Coulter
	PC5-conjugated anti-CD56	Beckman Coulter
	FITC-conjugated anti-CD14	Beckman Coulter
	FITC-conjugated anti-CD20	Biolegend
	APC/Cy7-conjugated IgG1	Biolegend
	BV510-conjugated IgG1	Biolegend
	BV421-conjugated mouse IgM	Becton-Dickinson
2	Anti-NKG2A (Z199) [IgG2b]	A. Moretta, S. Parolini
	Anti-KIR2 DL1/S1 (AZ115) [IgG1]	A. Moretta, S. Parolini
	Anti-KIR2 DL2/S2 (GL183) [IgG1]	A. Moretta, S. Parolini
	Anti-KIR3D (AZ158) [IgG2a]	A. Moretta, S. Parolini
	PE-conjugated goat-anti-mouse IgG1	Southern Biotech
	PE-conjugated goat anti-mouse IgG2a	Southern Biotech
	PE/Cy7-conjugated goat anti-mouse IgG2b	Southern Biotech
	FITC-conjugated anti-CD3	Beckman Coulter
	PC5-conjugated anti-CD56	Beckman Coulter
	FITC-conjugated anti-CD14	Beckman Coulter
	FITC-conjugated anti-CD20	Biolegend
	APC/Cy7-conjugated anti-CD16	Biolegend
	BV510-conjugated anti-CD69	Biolegend
	BV421-conjugated anti-CD57	Becton-Dickinson
3	Anti-SIGLEC7 (Z176) [IgG2b]	A. Moretta, S. Parolini
	Anti-LIR1 (F278) [IgG1]	A. Moretta, S. Parolini
	PE-conjugated goat-anti-mouse IgG1	Southern Biotech
	PE/Cy7-conjugated goat anti-mouse IgG2b	Southern Biotech
	FITC-conjugated anti-CD3	Beckman Coulter
	PC5-conjugated anti-CD56	Beckman Coulter
	FITC-conjugated anti-CD14	Beckman Coulter
	FITC-conjugated anti-CD20	Biolegend
	APC/Cy7-conjugated anti-CD16	Biolegend
	BV510-conjugated anti-CD69	Biolegend
	BV421-conjugated anti-CD57	Becton-Dickinson
4	Anti-NKG2C [IgG2b]	R&D Systems
	Anti-KIR2 DL1/S1 (11PB6) [IgG1]	A. Moretta, S. Parolini
	Anti-KIR2 DL2/S2 (GL183) [IgG1]	A. Moretta, S. Parolini
	Anti-KIR3D (AZ158) [IgG2a]	A. Moretta, S. Parolini
	PE-conjugated goat-anti-mouse IgG1	Southern Biotech
	PE-conjugated goat anti-mouse IgG2a	Southern Biotech
	PE/Cy7-conjugated goat anti-mouse IgG2b	Southern Biotech
	FITC-conjugated anti-CD3	Beckman Coulter
	PC5-conjugated anti-CD56	Beckman Coulter
	FITC-conjugated anti-CD14	Beckman Coulter
	FITC-conjugated anti-CD20	Biolegend
	APC/Cy7-conjugated anti-CD16	Biolegend
	BV510-conjugated anti-CD69	Biolegend
	BV421-conjugated anti-CD57	Becton-Dickinson
5	Anti-CXCR1 [IgG1]	Santa Cruz
	PE-conjugated goat-anti-mouse IgG1	Southern Biotech
	FITC-conjugated anti-CD3	Beckman Coulter
	PC5-conjugated anti-CD56	Beckman Coulter
	FITC-conjugated anti-CD14	Beckman Coulter
	FITC-conjugated anti-CD20	Biolegend
	APC/Cy7-conjugated anti-CD16	Biolegend
	Pacific Blue-conjugated anti-CCR7	Biolegend

### Analysis of Perforin Expression

Intracellular content of perforin was analyzed in a limited number of patients with SCID/OS/AS due to RAG/NHEJ defects and in healthy infants. Briefly, PBMCs were first stained with a mixture of FITC-conjugated mAbs directed against CD3, CD20, and CD14, as well as with PC5-labeled anti-CD56 mAb, and incubated for 30 min at 4°C. After treatment with 200 µl of Cytofix/Cytoperm (BD-Bioscience, Pharmigen CA, USA) for 20 min at 4°C, cells were washed with 1 ml of saponin (0.1% solution in PBS), and then stained with 5 µl of purified R-PE-labeled anti-perforin mAb (Ancell). After washing, the proportion of CD3^−^ CD14^−^ CD20^−^ CD56^+^ cells expressing perforin and the mean fluorescent intensity (MFI) of perforin were immediately analyzed on LSR Fortessa Flow Cytometer (BD) using FACSDiva software (BD). Final analysis was done using FloJo v.10.2 (TreeStar).

### Analysis of NK Cell Degranulation

Natural killer cell degranulation activity was tested against the K562 erythroleukemia human cell line. In particular, PBMCs derived from patients and from healthy donors were obtained upon Ficoll separation of heparinized blood samples, and incubated with or without 100 U/mL recombinant human IL-2 (NIH) at 37°C overnight. Cells were then incubated with target cells at an effector:target ratio of 1:3 in a final volume of 200 µl in round-bottomed 96-well plates at 37°C and 5% CO2 for 4 h in culture medium supplemented with anti-CD107a-PE (BD Biosciences Pharmingen, San Diego, CA, USA) monoclonal antibody. Cells were then surface-stained with FITC anti-CD3, PC5 anti-CD56, FITC anti-CD14 (Beckman Coulter), FITC anti-CD20, and APC/Cy7 anti-CD16 (BD Biosciences Pharmingen, San Diego, CA, USA) Ab for 30 min at 4°C. The cells were washed, and the proportion of CD3^−^ CD14^−^ CD20^−^ CD56^+^ cells expressing CD107a was analyzed immediately on LSR Fortessa Flow Cytometer (BD) using FACSDiva v6.1.3 software (BD Biosciences, Mountain View, CA, USA). Final analysis was performed using FloJo v.10.2 (TreeStar). The threshold to define CD107a expression in cells co-cultured with K562 target cells (in the presence or absence of IL-2) was set up on cells cultured with IL-2 alone, without K562 cells.

### Analysis of Interferon-γ (IFN-γ) Production

To detect intracellular production of IFN-γ, PBMCs from patients and healthy donors were incubated overnight at 37°C with IL-12 (0.5 ng/ml), or IL-12 (0.5 ng/ml) and IL-18 (0.1 ng/ml) combined. Surface staining was done by incubating the cells with FITC anti-CD3, PC5 anti-CD56, FITC anti-CD14 (Beckman Coulter), FITC anti-CD20, and APC/Cy7 anti-CD16 (BD) mAbs for 30 min at 4°C. Cells were then washed, fixed, and permeabilized with BD Cytofix/Cytoperm kit (BD Biosciences Pharmingen). IFN-γ production was detected by subsequent intracellular staining with PE-conjugated anti-IFN-γ (BD Biosciences Pharmingen). After washing, the proportion of CD3^−^ CD14^−^ CD20^−^ CD56^+^ cells expressing IFN-γ was immediately analyzed on LSR Fortessa Flow Cytometer (BD) using FACSDiva software (BD). Final analysis was done using FloJo v.10.2 (TreeStar).

### Statistical Analysis

Statistical analysis of the *in vitro* recombination activity of mutant RAG and ARTEMIS proteins, and of the expression of NK cell markers in patients and controls was performed using Mann–Whitney test for non-parametric variables.

## Results

### Demographic and Clinical Features

At the time of evaluation, mean age (±SEM) was significantly lower in patients with SCID (7.1 ± 1.72 months; range: 0.25–28 months) and in those with OS/AS (15.96 ± 5.41 months; range: 24–480 months) than in patients with CID (177.2 ± 28.22 months; range: 24–480 months; *p* < 0.0001).

A clinical history of significant infections was present in 60 patients with RAG/NHEJ gene defects (Table 1). Candida (*n* = 11), *Pseudomonas* (*n* = 7), human papillomavirus (HPV, *n* = 7), adenovirus (*n* = 6), varicella zoster virus (VZV, *n* = 6), *Pneumocystis jiroveci* (*n* = 5), Bacillus Calmette-Guerin (BCG, *n* = 5), and rhinovirus (*n* = 5) were the most common pathogens. In particular, *P. jiroveci* pneumonia was observed only in patients with SCID or OS/AS, whereas severe VZV infection was mostly restricted to patients with CID. Cytomegalovirus (CMV) and Epstein–Barr virus (EBV) infections were documented in 13 patients each, but were clinically significant only in 4 and 3 patients, respectively. Two SCID patients (P4 and P11) were diagnosed at birth following newborn screening, and infections were effectively prevented with isolation and prophylactic antimicrobials.

Autoimmunity and/or autoantibody production were documented in 23 patients, and were more common in patients presenting with CID (14 out of 21 patients) than in those with SCID (0/19) or with OS/AS (9/24). Cytopenias were the most frequent manifestation of autoimmunity and were documented in 11 patients (4 with OS/AS and 7 with CID). Alopecia and/or vitiligo were observed in six CID patients. Granulomatous disease was present in eight patients with CID. Finally, 12 patients (P20–P31) had typical features of OS (generalized erythroderma, lymphadenopathy, hepatosplenomegaly).

### Immunological Phenotype

**Figure 1:**
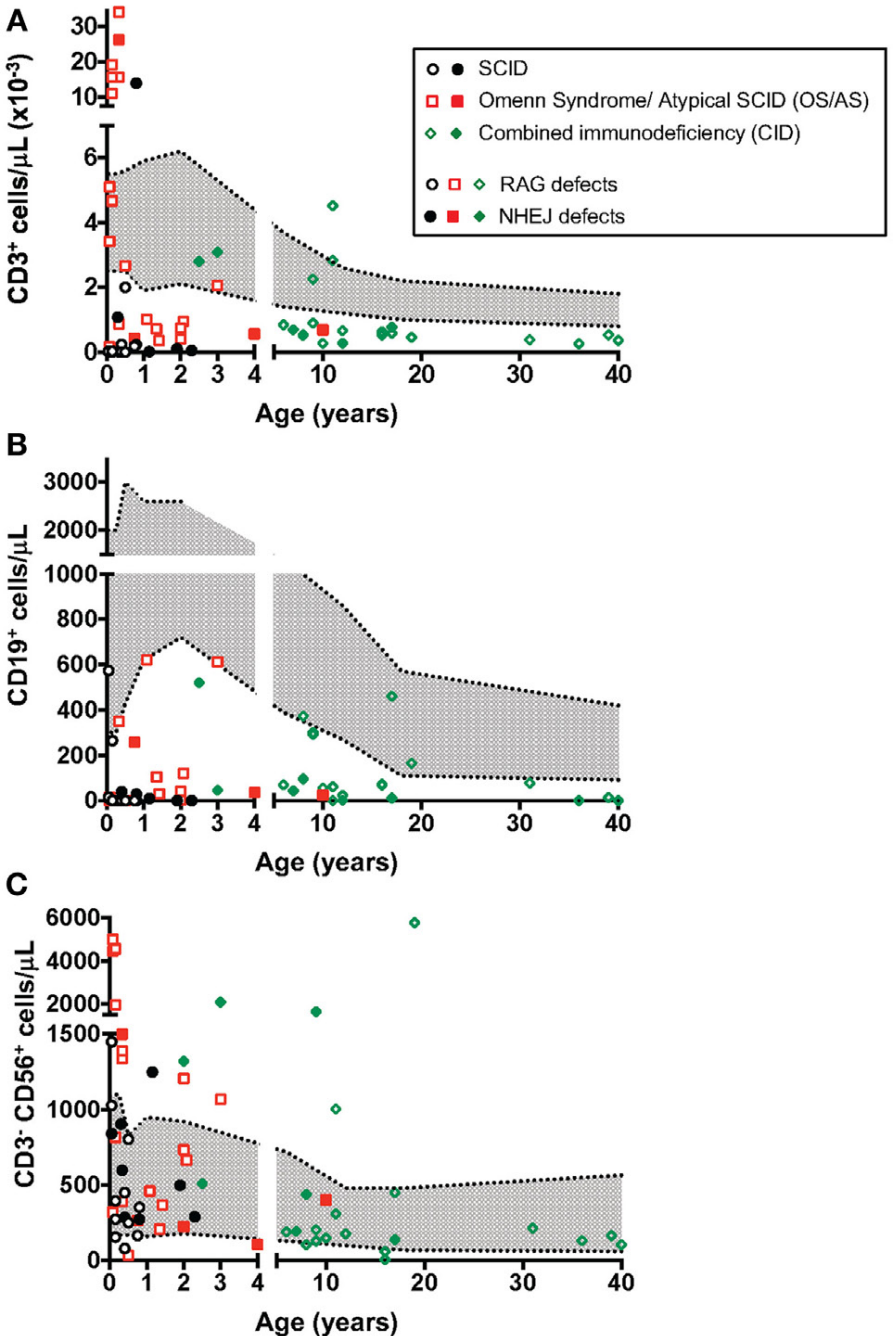
Absolute count of T **(A)**, B **(B)**, and natural killer (NK) **(C)** lymphocytes at the time of evaluation of NK cell phenotype and function. The area shown in shaded gray represents normal values for age. Different symbols (circle, square, diamond) are used to indicate patients with severe combined immune deficiency (SCID), Omenn syndrome/atypical SCID (OS/AS), and delayed onset combined immune deficiency (CID), respectively. Open circles identify patients with mutations of the recombinase-activating gene (RAG), and close symbols identify patients with mutations in genes involved in non-homologous end joining (NHEJ).

The count of circulating CD3^+^, CD19^+^, and CD3^−^ CD56^+^ cells in patients belonging to the various groups (SCID, OS/AS, and CID) is shown in Figure 1. With the exception of one SCID patient (P19) who had a high count of maternal T cells (13997 cells/μl), all others had severe T cell lymphopenia (Figure 1A). By contrast, a higher count of autologous T cells was recorded in patients with CID and especially in patients with OS/AS. B cell lymphopenia was observed in the vast majority of patients, irrespective of the clinical phenotype (Figure 1B). By contrast, NK cell count was either normal or increased in most patients (Figure 1C).

Data on *in vitro* proliferation to phytohemagglutinin (PHA) were available in 51 patients. Response was absent (<10% of lower limit of normal, LLN) in 17, markedly reduced (10–30% of LLN) in 11, reduced (>30% of LLN, but lower than LLN) in 14, and normal in 9 patients. T cell proliferation to PHA was better preserved in patients with CID; among 18 such patients for whom information was available, 15 had either normal (*n* = 7) or reduced (*n* = 8) response.

Low serum levels of IgA and IgM were detected in the majority of SCID patients (Table 1); serum IgG in this group was less informative because of the presence of maternally derived immunoglobulins. Immunoglobulin levels were more variable in patients with OS/AS and in those with CID. In particular, among 23 CID patients, 17 had either low serum IgG (*n* = 9) or were under immunoglobulin replacement therapy (*n* = 8) and 15 had undetectable serum IgA.

### Correlation between Clinical Phenotype and Recombination Activity Sustained by the Mutant Alleles

**Figure 2:**
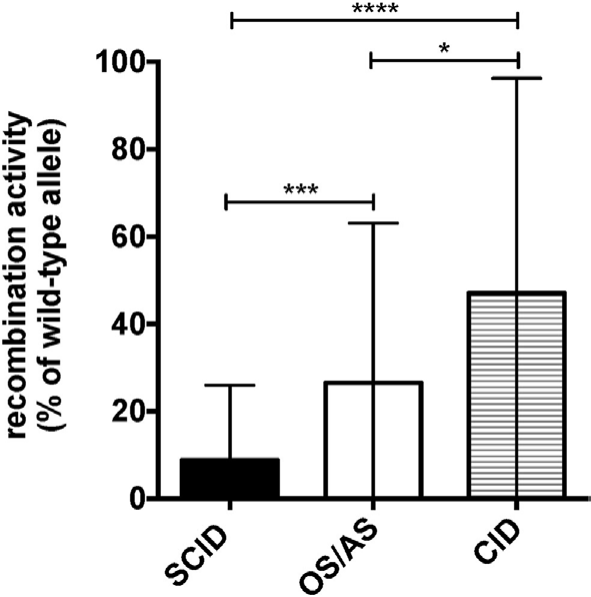
Graphical representation of the recombination activity (mean ± SD) of *RAG* and *DCLRE1C* mutant alleles in patients with severe combined immune deficiency (SCID), Omenn syndrome/atypical SCID (OS/AS), and delayed onset combined immune deficiency (CID). *p < 0.05; ***p < 0.005; ****p < 0.001.

Molecular analysis of the patients included in this study identified a total of 41 *RAG1*, 16 *RAG2*, 11 *DCLRE1C*, 4 *LIG4*, and 2 *NHEJ* distinct gene mutations (Table 1). Using a flow cytometry-based assay, we have previously reported correlation between recombination activity supported by *RAG* and *DCLRE1C* mutant alleles and the clinical and immunological disease phenotype (22, 32). For the patient cohort included in this study, data on *in vitro* recombination activity were available for 36 *RAG1*, 9 *RAG2*, and 6 *DCLRE1C* mutant alleles. Upon plotting for each patient the recombination activity associated with each of the two mutant alleles, a significant genotype–phenotype correlation was observed, with higher residual recombination activity for mutant alleles associated with a CID phenotype than for those associated with OS/AS or SCID (Figure 2).

### Circulating NK Cells from Healthy Infants Comprise a High Proportion of CD56^bright^ Cells

**Figure 3:**
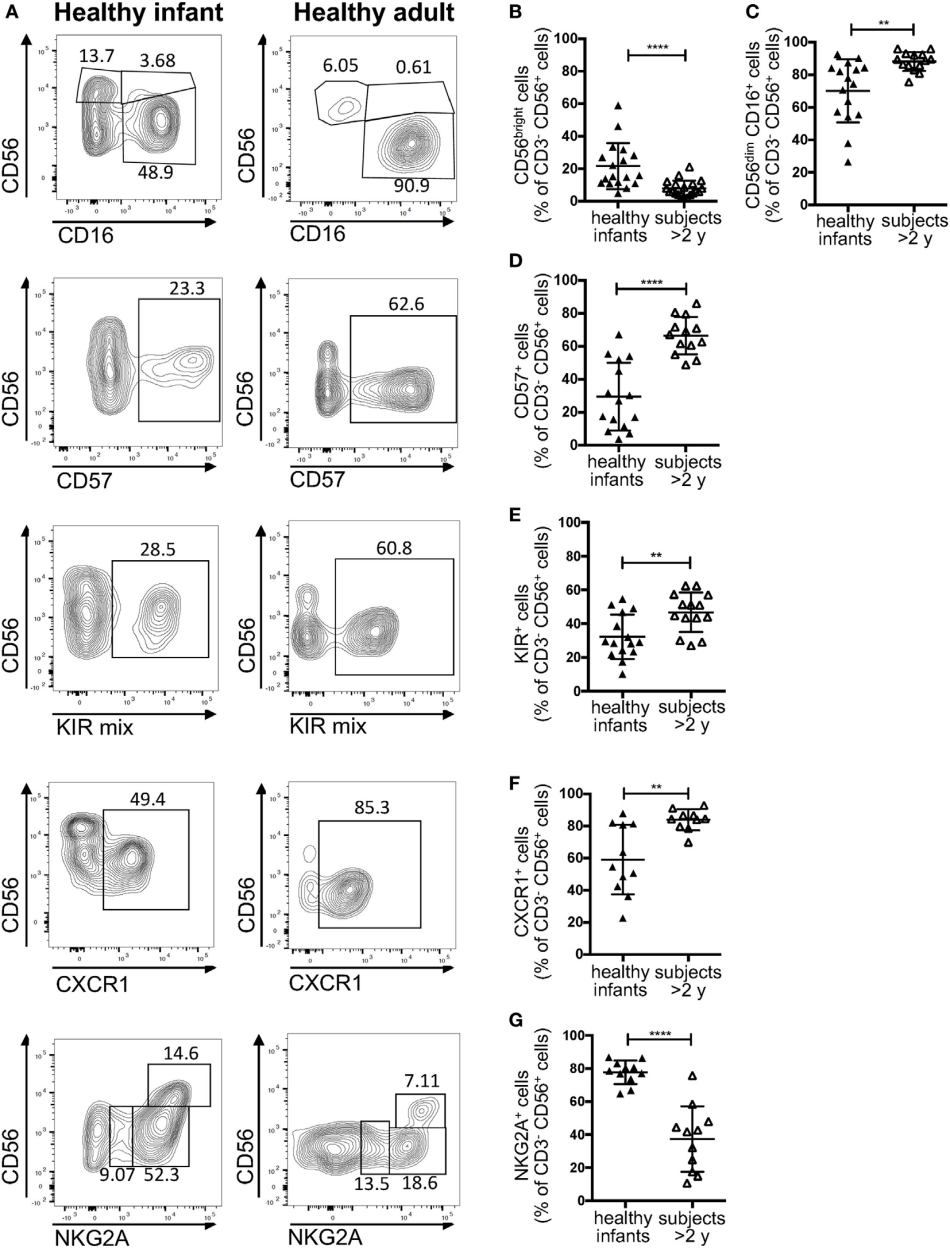
Flow cytometry analysis of natural killer cell phenotype in healthy infants (<2 years of age) and healthy donors >2 years of age. **(A)** Representative analysis of the expression of CD56, CD16, CD57, killer cell immunoglobulin-like receptors (KIRs), CXCR1, and NKG2A in a healthy infant and a healthy donor >2 years of age; **(B–G)** percentage of CD56^bright^ **(B)**, CD16^+^ **(C)**, CD57^+^ **(D)**, KIR^+^ **(E)**, CXCR1^+^ **(F)**, and NKG2A^+^ **(G)** cells upon gating on CD3^−^ CD19^−^ CD14^−^ CD56^+^ cells. For NKG2A, different gates are shown to indicate NKG2A^+^, NKG2A^++^, and NKG2A^+++^ cells. For each marker, positivity was defined as fluorescence intensity above that of isotype control. Bars represent mean ± SD values. **p < 0.01; ****p < 0.001.

According to a widely accepted scheme, human NK cell differentiation is marked by changes in the expression of cell surface and intracytoplamsic markers, so that five distinct stages of NK cell development are recognized (33). In peripheral blood from healthy controls, both stage 4 (CD56^bright^ CD16^−^) and stage 5 (CD56^+^ CD16^hi^) NK cells are detectable. In particular, in normal adults, CD56^bright^ CD16^−^ cells account for only 5–10% of all circulating NK cells. By contrast, a higher percentage of CD56^bright^ cells was detected in healthy infants (Figures 3A,B). Moreover, the proportion of CD16^+^ cells was lower in infants than in healthy controls greater than 2 years of age (Figures 3A,C). Finally, NK cells from healthy infants included a lower proportion of cells expressing CD57 (Figures 3A,D), KIRs (Figures 3A,E), and CXCR1 (Figures 3A,F), whereas the percentage of NK cells expressing NKG2A was higher in infants than in older healthy controls (Figures 3A,G).

### Markedly Increased Proportion of CD56^bright^ NK Cells in the Peripheral Blood of Patients with RAG and NHEJ Defects Correlates with the Clinical Phenotype

**Figure 4:**
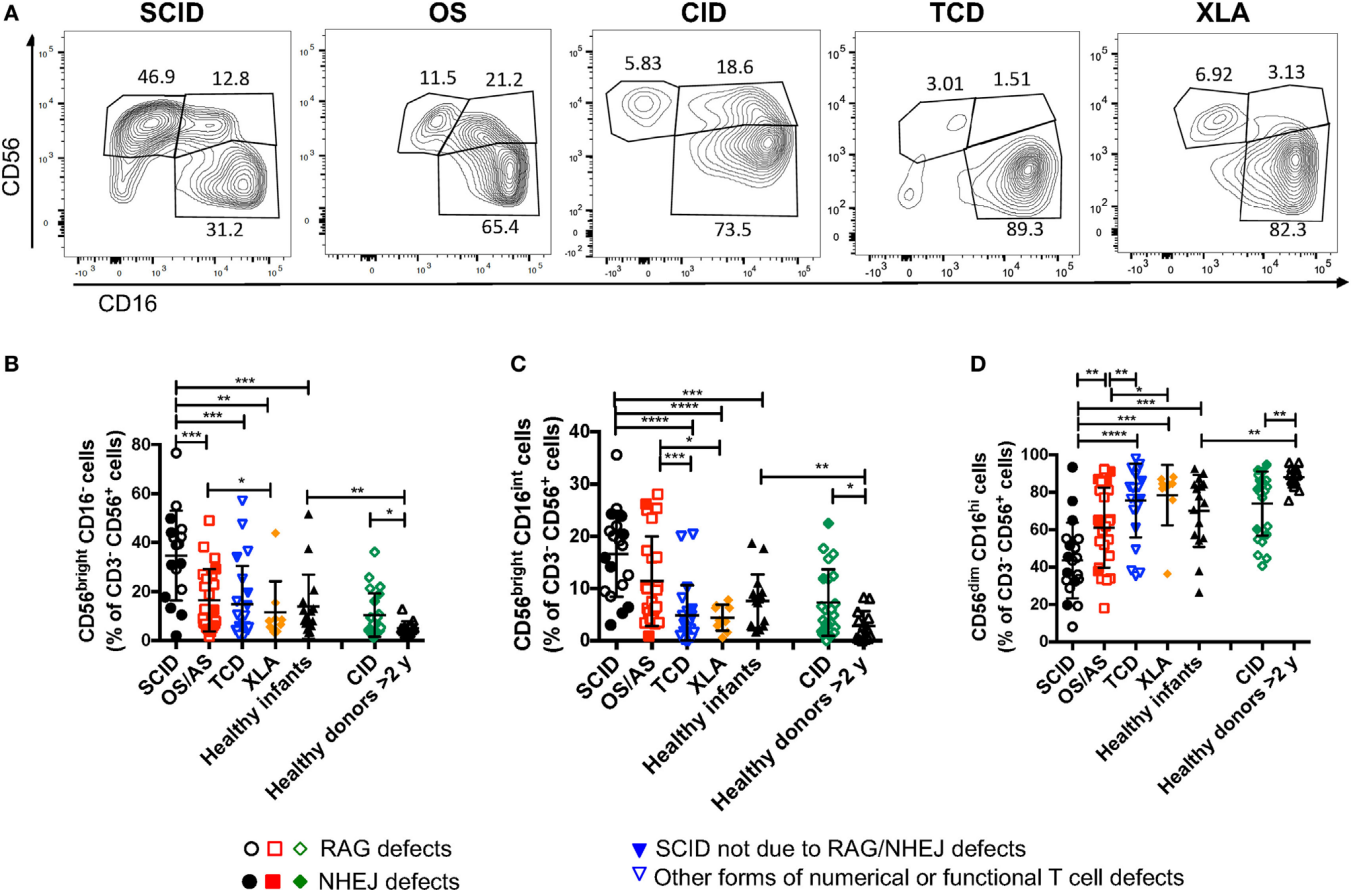
Flow cytometry analysis of CD56 and CD16 expression in natural killer cells from patients and controls. **(A)** Representative analysis of the expression of CD56 and CD16 in CD3^−^ CD19^−^ CD14^−^ CD56^+^ cells from one patient each belonging to the severe combined immune deficiency (SCID), Omenn syndrome (OS), delayed-onset combined immune deficiency (CID), other forms of T cell defect (TCD), and X-linked agammaglobulinemia (XLA), respectively. **(B–D)** Percentage of CD56^bright^ CD16^−^ **(B)**, CD56^bright^ CD16^int^ **(C)**, and CD56^dim^ CD16^hi^ **(D)** cells among CD3^−^ CD19^−^ CD14^−^ CD56^+^ cells. For each marker, positivity was defined as fluorescence intensity above that of isotype control. Bars represent mean ± SD values. *p < 0.05; **p < 0.01; ***p < 0.005; ****p < 0.001.

A recent study in *Rag^−/−^* mice has unexpectedly disclosed abnormalities of NK phenotype and function, with increased proportion of mature, activated cells with increased cytotoxic activity and reduced survival (30). In order to assess whether similar abnormalities exist in patients with RAG or NHEJ defects, and to establish whether such abnormalities, if present, correlate with the severity of the clinical phenotype and with residual function of the mutant protein, we have analyzed the expression of CD56 and CD16 on the surface of CD3^−^ CD14^−^ CD20^−^ peripheral blood NK cells from patients and controls. As shown in Figures 4A,B, patients with SCID due to RAG/NHEJ defects had a high proportion of CD56^bright^ CD16^−^ NK cells (mean ± SEM: 34.7 ± 4.4), that exceeded what was observed in healthy infants (13.9 ± 3.3; *p* = 0.0007). Similarly, the proportion of CD56^bright^ CD16^−^ NK cells was higher in patients with CID than in healthy controls greater than 2 years of age (10.4 ± 1.8 vs. 5.1 ± 0.7; *p* = 0.0431; Figures 4A,B). Among infants with RAG/NHEJ defects, the proportion of CD56^bright^ CD16^−^ NK cells was higher in those presenting with SCID than in those with OS/AS (*p* = 0.0008; Figure 4B). In order to investigate whether the increase in the proportion of CD56^bright^ NK cells was secondary to numerical and/or functional abnormalities of T and/or B cells, we also analyzed a group of patients with other forms of TCD or with XLA. The proportion of CD56^bright^ CD16^−^ NK cells in these groups was similar to that of healthy controls, and significantly lower than in patients with SCID due to RAGH/NHEJ defects (Figure 4B). Among patients with TCD, no correlation was observed among the proportion of CD56^bright^ NK cells and the severity of T cell lymphopenia, indicating that the increase of CD56^bright^ NK cells observed in SCID patients with RAG/NHEJ defects is not simply secondary to T cell lymphopenia. Furthermore, the proportion of CD56^bright^ CD16^int^ NK cells, that are considered to represent an intermediate step of differentiation between CD56^bright^ CD16^−^ and CD56^dim^ CD16^hi^ cells, was significantly higher in infants with SCID due to RAG/NHEJ defects than in healthy infants, patients with TCD or with XLA (*p* < 0.0001; Figure 4C). A higher percentage of CD56^bright^ CD16^int^ NK cells was also observed in patients with CID than in healthy controls greater than 2 years of age (*p* < 0.05; Figure 4C). Finally, patients with SCID due to RAG/NHEJ defects had a lower proportion of CD56^dim^ CD16^hi^ NK cells when compared to healthy infants (*p* = 0.0007; Figure 4D). Similar results were obtained when comparing patients with CID versus healthy controls older than 2 years of age (*p* = 0.0011).

### CD56^bright^ NK Cells from Infants with RAG/NHEJ Defects Have an Immature Phenotype

**Figure 5:**
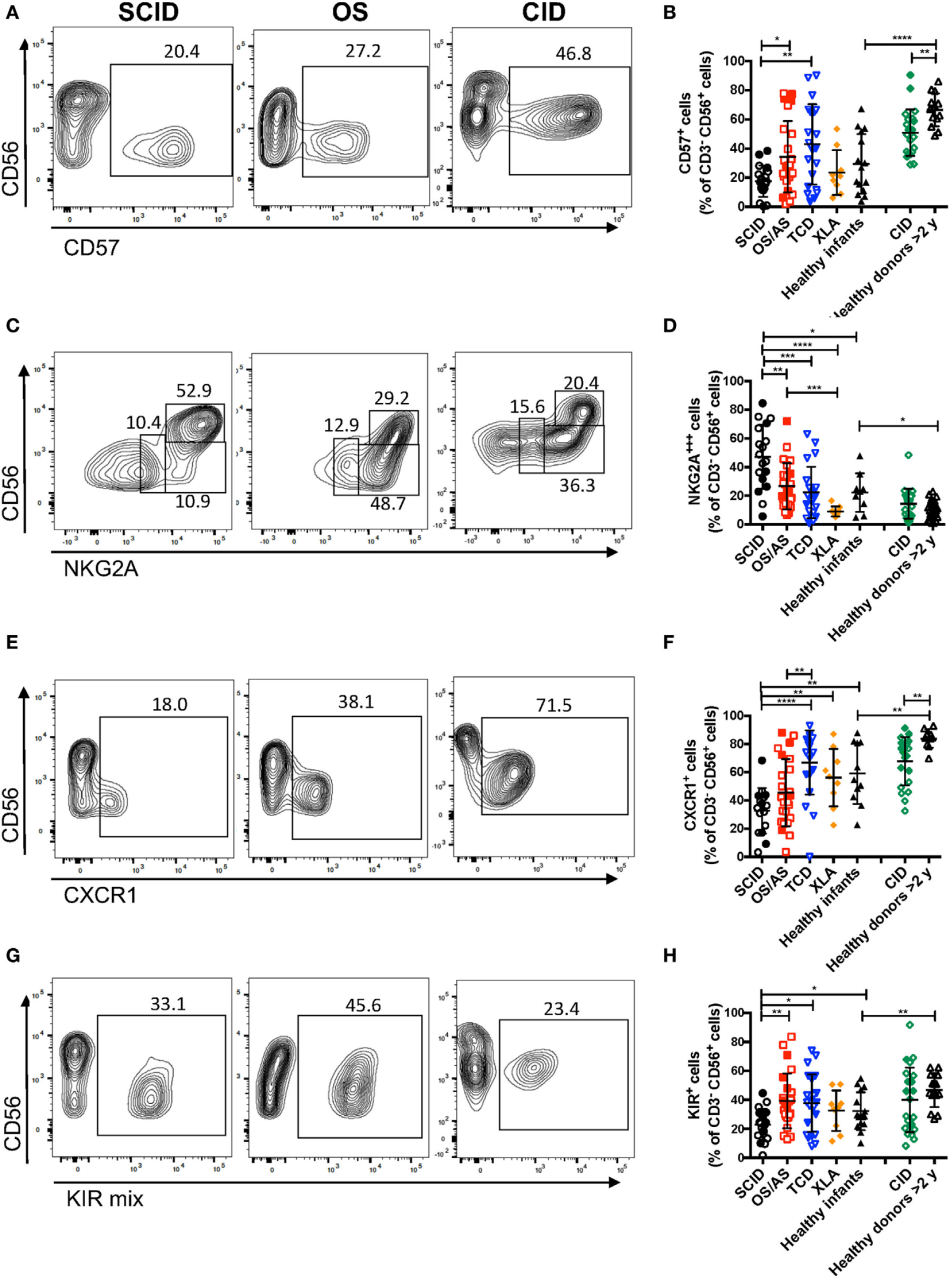
Flow cytometry analysis of the expression of CD57 **(A,B)**, NKG2A **(C,D)**, and CXCR1 **(E,F)**, and killer cell immunoglobulin-like receptors (KIRs) **(G,H)** among CD3^−^ CD19^−^ CD14^−^ CD56^+^ cells. In panels **(A,C,E,G)**, representative examples are shown for one patient each belonging to the severe combined immune deficiency (SCID), Omenn syndrome (OS), and delayed-onset combined immune deficiency (CID) subgroups. For each marker, positivity was defined as fluorescence intensity above that of isotype control. Bars represent mean ± SD values. *p < 0.05; **p < 0.01; ***p < 0.005; ****p < 0.001.

The data reported above on CD56 and CD16 surface expression indicate that patients with RAG/NHEJ defects have an abnormal distribution of NK cell subsets, with an increased proportion of more immature cells as compared to what is observed in healthy controls. In order to further confirm this, we analyzed the expression of additional surface markers that are differentially expressed in CD56^bright^ versus more mature CD56^dim^ NK cells. As compared to healthy controls >2 years of age, patients with CID had a lower proportion of CD57^+^ NK cells (*p* = 0.0022; Figures 5A,B). Furthermore, patients with SCID had fewer CD57^+^ NK cells than patients with other forms of TCD (*p* = 0.0041), and a similar trend was also observed when compared to infant controls, although the difference did not reach statistical significance (Figure 5B).

**Figure 6:**
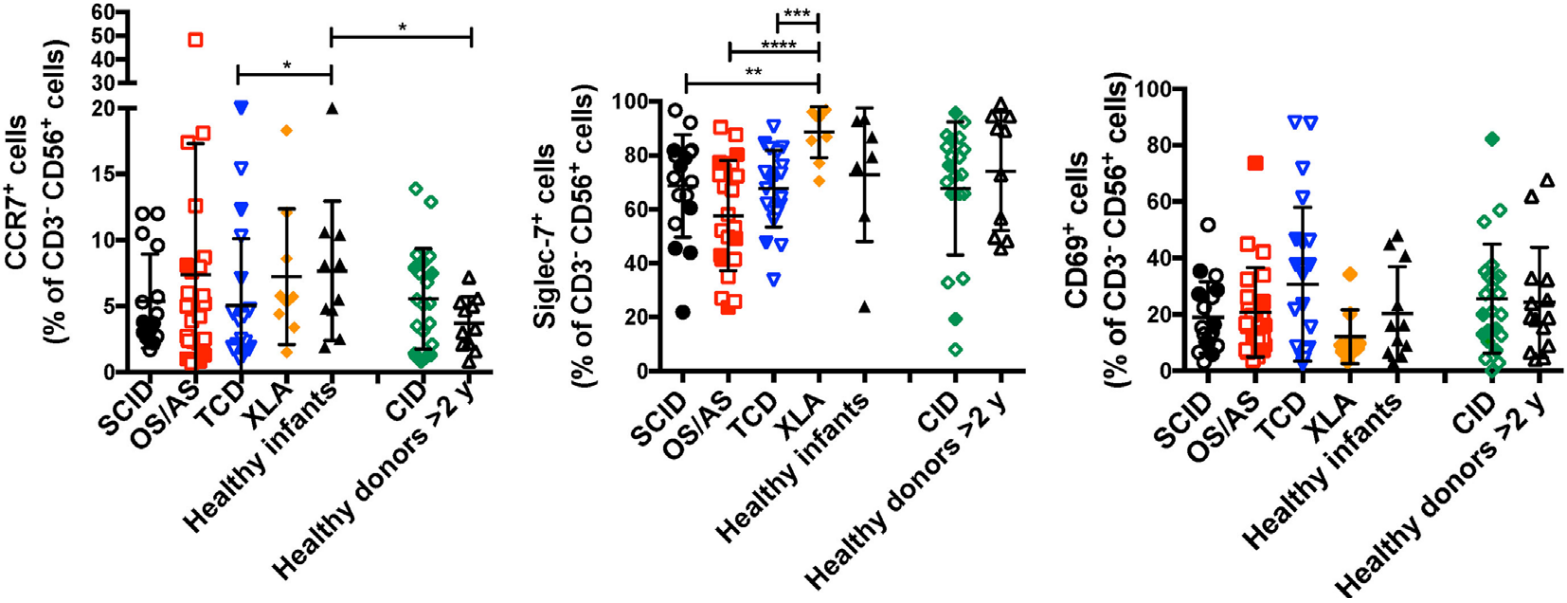
Percentage of CD3^−^ CD19^−^ CD14^−^ CD56^+^ cells expressing CCR7 (left panel), Siglec-7 (middle panel), or CD69 (right panel) in patients and controls. For each marker, positivity was defined as fluorescence intensity above that of isotype control. Bars represent mean ± SD values. *p < 0.05; **p < 0.01; ***p < 0.005; ****p < 0.001.

In normal subjects, circulating stage 4 CD56^bright^ NK cells are characterized by high levels of expression of NKG2A and of the chemokine receptor CCR7, which facilitates homing to lymphoid tissues. By contrast, expression of KIRs and of the chemokine receptor CXCR1 is virtually absent in CD56^bright^ NK cells. As compared to healthy controls, the proportion of NK cells expressing NKG2A at high density was increased in patients with SCID and CID due to RAG/NHEJ defects (Figures 5C,D), and by contrast, the percentage of CXCR1^+^ NK cells was reduced (Figures 5E,F). Furthermore, patients with SCID due to RAG/NHEJ defects had a lower proportion of NK cells expressing KIR than healthy infants, patients with OS and those with other forms of TCD (Figures 5G,H). While the proportion of CCR7^+^ cells was higher in healthy infants than in healthy controls >2 years of age (Figure 6), no significant differences were observed in the percentage of CCR7^+^ NK cells between patients with RAG/NHEJ defects and healthy controls. Notably, CCR7 expression was downregulated during the transition from CD56^bright^ CD16^−^ to CD56^bright^ CD16^int^ cells (data not shown). Furthermore, the proportion of NK cells expressing Siglec-7, leukocyte Ig-like receptor (LIR)-1, or the activation marker CD69 was similar in patients and healthy controls, although interestingly a high proportion of Siglec-7^+^ NK cells were detected in the control group of patients with XLA (Figure 6). Overall, these data support the notion that the CD56^bright^ NK cells that are more abundant in patients with RAG/NHEJ defects represent immature NK cells and provide evidence for a genotype–phenotype correlation in this group of diseases.

### Impact of Viral Infections on NK Cell Phenotype

**Figure 7:**
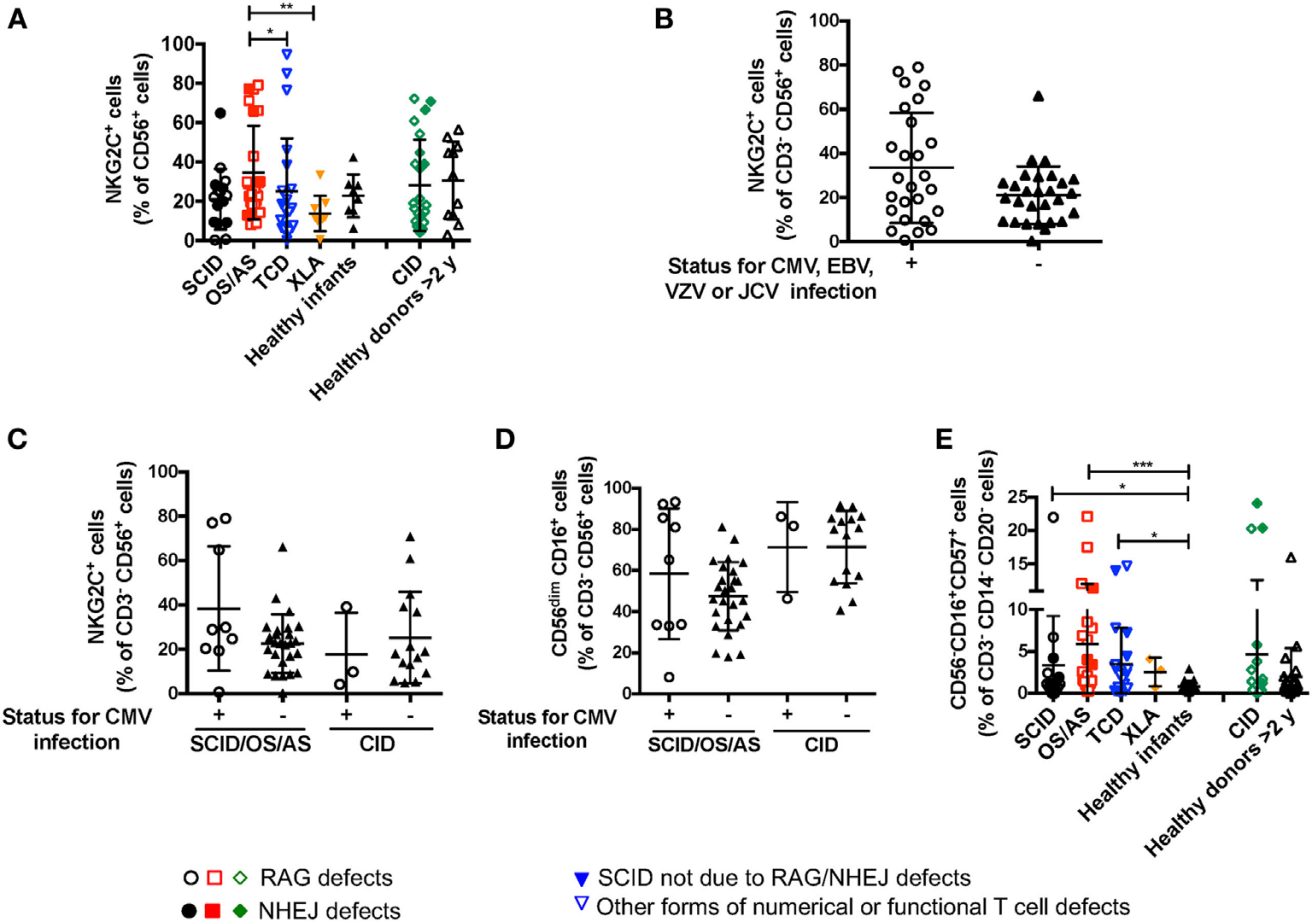
**(A)** Percentage of NKG2C^+^ cells among CD3^−^ CD19^−^ CD14^−^ CD56^+^ cells from patients and controls. **(B)** Percentage of NKG2C^+^ cells among natural killer (NK) cells from patients with recombinase-activating gene (RAG)/non-homologous end-joining (NHEJ) defects with positive (+) or negative (−) history of infections due to cytomegalovirus (CMV), Epstein–Barr virus (EBV), varicella zoster virus (VZV), or JC virus (JCV). **(C)** Percentage of NKG2C^+^ cells among NK cells from patients with RAG/NHEJ defects presenting with severe combined immune deficiency (SCID), Omenn syndrome/atypical SCID (SCID/OS/AS), or with delayed onset combined immune deficiency (CID), and divided into two groups according to the presence (+) or absence (−) of a history of CMV infection. **(D)** Percentage of CD56^dim^ CD16^+^ cells among NK cells from patients with RAG/NHEJ defects presenting with SCID/OS/AS or with CID, and with either positive (+) or negative (−) history of CMV infection. **(E)** Percentage of CD56^−^ CD16^+^ CD57^+^ cells among CD3^−^ CD14^−^ CD20^−^ peripheral blood mononuclear cells. Bars represent mean ± SD values. *p < 0.05; **p < 0.01; ***p < 0.005.

Recombinase-activating gene and NHEJ defects lead to increased susceptibility to viral infections, including CMV, EBV, and VZV. It has been reported that viral infections (and CMV in particular) may lead to significant changes of NK cell phenotype and function, with accumulation of cytotoxic CD56^dim^ NKG2C^+^ NKG2A^−^ KIR^+^ CD57^+^ cells (34). Expansion of these cells does not require T cells, as shown in an IL7R-deficient SCID infant after CMV infection (35). A significant proportion (26/66, 39.4%) of the patients with RAG/NHEJ defects included in this study had a documented history of CMV, EBV, VZV or severe JC virus (JCV) infection (Table 1). The proportion of NK cells expressing NKG2C was not statistically different in patients with SCID, OS/AS, or CID versus healthy controls (Figure 7A). Furthermore, when patients were divided into two groups according to their history of CMV, EBV, VZV, or severe JCV infection, no differences were observed in the proportion of NKG2C^+^ NK cells (Figure 7B). Even when patients with SCID/OS/AS or with CID were divided into two subgroups based on their CMV infection history, no difference was observed in the proportion of NKG2C^+^ (Figure 7C) and of CD56^dim^ (Figure 7D) NK cells. Finally, although a higher percentage of “exhausted” NK cells with a CD56^−^ CD16^+^ CD57^+^ phenotype was detected in patients with SCID, OS/AS, and TCD than in healthy infants (Figure 7E), no correlation was observed with a history of viral infections.

### CD56^bright^ Cells from Patients with RAG/NHEJ Defects Are Potent Producers of IFN-γ

**Figure 8:**
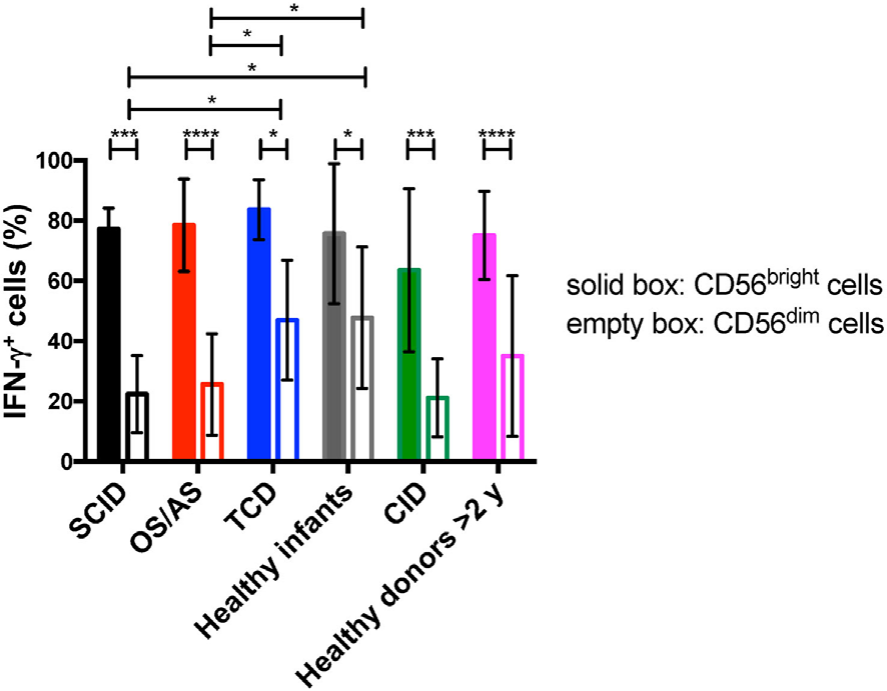
Percentage of interferon-γ (IFN-γ) expressing cells among CD56^bright^ and CD56^dim^ natural killer cells from patients and controls, upon *in vitro* stimulation with IL-12 and IL-18. Bars represent mean ± SD values. *p < 0.05; ***p < 0.005; ****p < 0.001.

Circulating CD56^bright^ NK cells serve an immunoregulatory function through secretion of various cytokines (IFN-γ, TNF-α, IL-10) (36). In order to assess this aspect of NK cell function, we performed *in vitro* stimulation with IL-12 and IL-18, and analyzed intracellular expression of IFN-γ upon gating separately on CD56^bright^ vs. CD56^dim^ NK cells. As expected, in both patients and controls CD56^bright^ cells were more potent producers of IFN-γ than CD56^dim^ cells, but both subsets were capable of producing this cytokine (Figure 8). However, CD56^dim^ cells from patients with SCID and with OS/AS due to RAG/NHEJ defects showed impaired IFN-γ production when compared to equivalent cells from healthy infants or patients with TCD. A similar trend was observed in patients with CID versus healthy controls greater than 2 years of age (Figure 8).

### NK Cells from Patients with SCID due to RAG/NHEJ Defects Have Increased Degranulation Capacity in the Absence of IL-2-Mediated Stimulation, and Express a Higher Amount of Intracellular Perforin than NK Cells from Healthy Infant Controls

**Figure 9:**
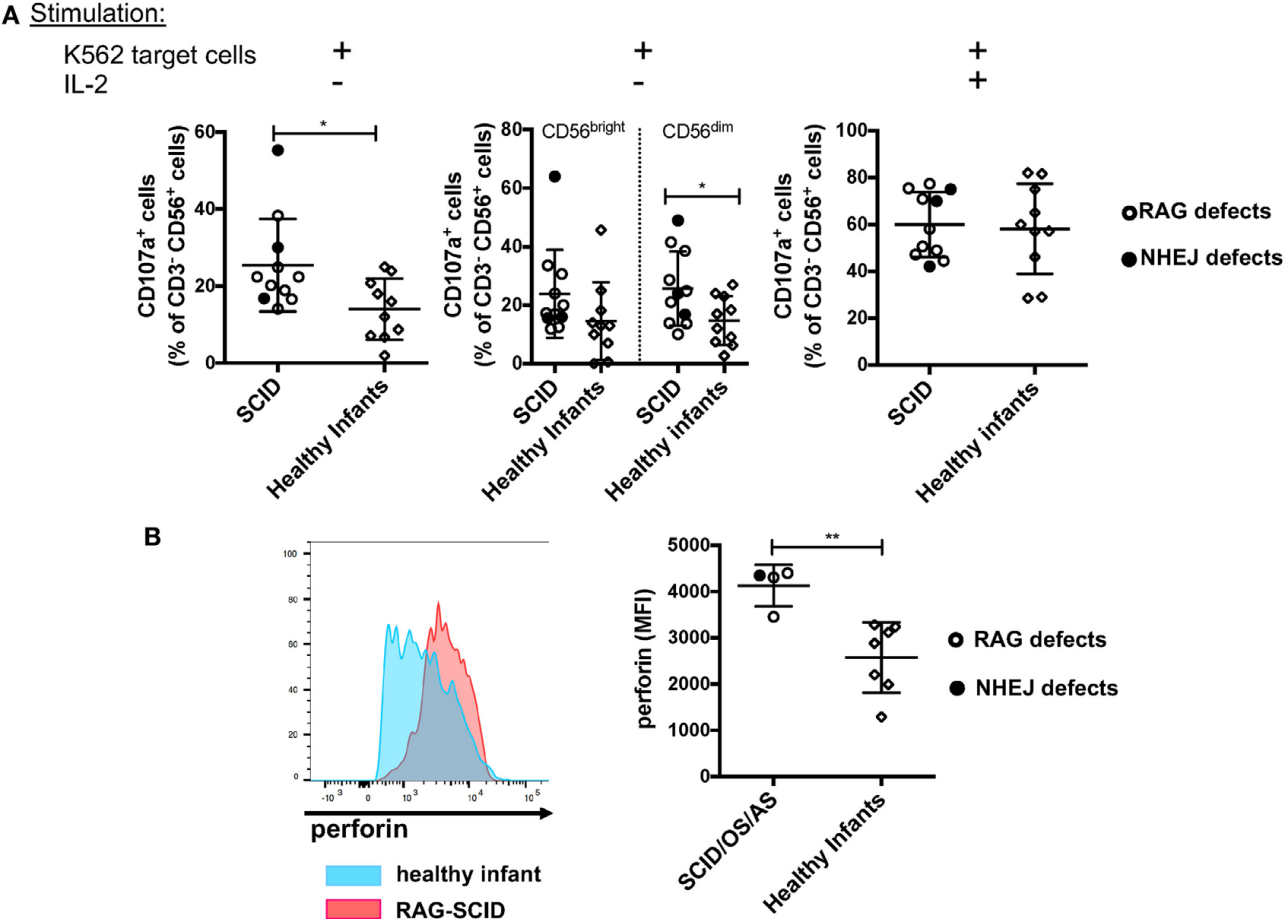
**(A)** Natural killer (NK) cells degranulation, expressed as percentage of CD107a^+^ cells, upon co-culture of peripheral blood mononuclear cells from patients with severe combined immune deficiency (SCID) and healthy infants with K562 target cells, in the absence (left and middle panels) or presence (right panel) of stimulation with exogenous interleukin-2 (IL-2). The middle panel shows degranulation of CD56^bright^ (left) and CD56^dim^ (right) NK cells from SCID patients and healthy infants in the absence of IL-2 stimulation. **(B)** Representative example (left) of perforin expression in resting NK cells from a patient with SCID due to RAG defect (red) and a healthy infant (blue), and mean fluorescent intensity (MFI) of perforin expression (right) in patients with SCID/OS/AS due to recombinase-activating gene (RAG)/non-homologous end-joining (NHEJ) defects and healthy infants. Bars represent mean ± SD values. *p < 0.05; **p < 0.01.

Previous data had shown that NK cells from *rag^−/−^* mice have increased cytotoxic activity (30). To investigate the cytolytic machinery of NK cells, we have analyzed NK cell degranulation in response to K562 target cells, in the absence or presence of IL-2. It is well known that human CD56^bright^ NK cells have reduced cytotoxic activity when compared to CD56^dim^ cells (8). In spite of the markedly increased proportion of CD56^bright^ NK cells, we observed that in the absence of stimulation with IL-2, NK cells from patients with SCID due to RAG/NHEJ defects have increased degranulation capacity when compared to NK cells from healthy infants (Figure 9A, left panel). In particular, CD56^dim^ NK cells from patients with SCID showed increased degranulation when compared to the equivalent subset of NK cells from healthy infants (Figure 9A, middle panel). A similar trend was observed also for CD56^bright^ NK cells, but the difference between SCID patients and controls did not reach statistical significance (Figure 9A, middle panel). Stimulation with IL-2 significantly increased NK cell degranulation capacity to similar levels in patients and controls (Figure 9A, right panel). Although the amount of intracellular perforin could be measured only in a small number of patients with SCID, a significantly higher mean fluorescent intensity (MFI) was observed in unstimulated NK cells from patients than from healthy infants (Figure 9B).

## Discussion

In this manuscript, we have demonstrated that NK cells from healthy infants comprise a higher proportion of CD56^bright^ CD16^−/int^ CD57^−^ cells than what is observed in older healthy subjects, confirming previous observations (37–39). Several lines of evidence indicate that CD56^bright^ cells represent precursors to CD56^dim^ cells. In particular, it has been demonstrated that appearance of CD56^bright^ cells precedes that of CD56^dim^ cells after HSCT (40) and that it is possible to induce differentiation of CD56^bright^ into CD56^dim^ NK cells *in vitro* in response to signaling *via* fibroblast growth factor receptor 1 (41). On the other hand, the model according to which CD56^bright^ cells precede CD56^dim^ cells during NK cell development has been challenged by NK cell lineage tracking experiments with genetic bar coding in macaques, which have suggested that these subsets may have distinct ontogeny (42).

In any case, the demonstration that composition of the peripheral NK cell compartment varies with age indicates that appropriate age-matched controls should be used when analyzing NK cell phenotype in pathological conditions.

In particular, by performing an extensive phenotypic analysis, we have shown that NK cells from patients with RAG/NHEJ defects comprise a higher proportion of CD56^bright^ CD16^−/int^ NKG2A^+++^ cells, and a reduced percentage of CD56^dim^ CD16^hi^ cells expressing CD57, KIRs, and CXCR1, than observed in age-matched healthy controls. Altogether, these observations suggest that NK cells from patients with RAG/NHEJ defects have a more immature phenotype when compared to age-matched healthy controls, in spite of the fact that NK cells from CID patients showed signs of progressive differentiation as compared to NK cells from patients with SCID/OS/AS. These data contrast with recent findings in *Rag^−/−^* mice, whose peripheral NK cell compartment is characterized by a predominance of cells expressing KLRG1, a marker of mature NK cells (30). Furthermore, peripheral NK cells from *Rag^−/−^* mice are largely CD62L^−^, indicating an *in vivo* activated phenotype (30). By contrast, we did not observe increased expression of CD69, an activation marker (43), in NK cells from patients with RAG/NHEJ defects, including those with OS, a condition characterized by accumulation of *in vivo*-activated autologous T cells. While the reasons for these discrepancies are not clear, murine and human NK cells differ substantially, also in regard to phenotypic markers that are differentially expressed during development.

By affecting the process of V(D)J recombination, mutations in *RAG* and NHEJ genes profoundly affect generation of T and B lymphocytes. Consistent with this, patients with *RAG*/NHEJ gene defects had a markedly reduced number of circulating B and T cells, with the exception of patients with OS, some of which had a normal or even increased number of oligoclonal and activated T cells, as typically observed in this disease. Previous studies in TCRβδ*^−/−^* and in μMT*^−/−^* mice had demonstrated that lack of T and B cells have opposite effects on the count of splenic NK cells. In particular, T cell deficiency is associated with a twofold to threefold increase, and B cell deficiency with a twofold to threefold decrease of NK cell count as compared to C57BL/6 wild-type mice (44). The patients with RAG and NHEJ defects included in this study had either normal or increased count of circulating NK cells. In order to rule out the possibility that their abnormalities of NK cell phenotype could be secondary to T and B cell lymphopenia, we have included in this study patients with XLA (who lack circulating B cells) and with other forms of TCD, including seven patients with SCID not due to RAG/NHEJ defects. Even when compared to these two control groups, patients with SCID due to RAG/NHEJ gene defects had a markedly increased proportion of CD56^bright^ CD16^−^ CD57^−^ NKG2A^+++^ cells. Similar differences were also observed also when comparing NK cells from patients with OS/AS and those with TCD or XLA, with an increased percentage of CD56^bright^ CD16^−^ CD57^−^ cells in the former. Altogether, these data indicate that the increased representation of immature cells within the peripheral NK cell compartment of patients with RAG/NHEJ defects is not secondary to T and B cell lymphopenia.

Because of their severe T cell immunodeficiency, patients with RAG/NHEJ defects are highly prone to viral infections, including CMV. LIR-1 is a member of the immunoglobulin superfamily which has been shown to bind the human CMV MHC class I homolog UL-18 protein (45). NKG2A and NKG2C represent inhibitory and activating forms of CD94, recognizing non-classical HLA-E molecules (46). CMV infection can drive expansion of KIR^+^ and/or LIR-1^+^, NKG2A^−^ NKG2C^+^ NK cells (34). Furthermore, rapid differentiation of CD56^dim^ NKG2C^+^ NKG2A^−^ CD57^+^ cells has been observed after CMV reactivation in recipients of HSCT (47). It has been shown that upon transfer from CMV-seropositive donors into seropositive HSCT recipients, NKG2C^+^ cells undergo expansion and produce high amounts of IFN-γ as compared to NKG2C^+^ cells transfused into CMV-seronegative recipients, implying that NKG2C^+^ cells may represent “memory” NK cells capable of a prompt response upon re-exposure to CMV (48). Finally, rapid expansion of NKG2C^+^ NK cells has been reported in infants acquiring perinatal CMV infection (39). Although several of the patients included in this study had a history of CMV infection, no particular expansion of NKG2C^+^ and/or LIR-1^+^ cells was observed. Monocytes are apparently required to allow expansion of adaptive/memory-like NK cells (49). Lack of expansion of NKG2C^+^ cells in CMV-infected, RAG-/NHEJ mutated SCID patients may reflect a requirement also for T cell help in this process and may further contribute to poor control of CMV infection in these patients.

CD56^bright^ and CD56^dim^ NK cells differ in their immunomodulatory and effector function. In particular, CD56^bright^ cells are potent producers of IFN-γ, but have lower content of perforin and display reduced cytotoxic activity as compared to CD56^dim^ cells. Analysis of IFN-γ production by CD56^bright^ and CD56^dim^ NK cells from patients and controls confirmed that CD56^bright^ cells were more potent producers of IFN-γ, irrespective of the underlying diagnosis. However, the proportion of CD56^dim^ NK cells producing IFN-γ was lower in patients with SCID/OS/AS due to RAG/NHEJ defects than in healthy infants or in patients with other forms of TCD, and a similar trend was observed for CD56^dim^ cells from CID patients. These results suggest that RAG/NHEJ defects are associated with abnormalities of NK cells that are not restricted to their phenotype, but may also involve NK cell function. In this regard, it is particularly significant that NK cells from the patients displayed enhanced degranulation in the absence of previous stimulation with IL-2, and that CD56^bright^ cells from patients with SCID/OS/AS had a higher content of perforin than CD56^bright^ cells from healthy infants. Although we did not perform a chromium release assay to investigate NK cytolytic function, these data suggest that RAG/NHEJ defects are associated with enhanced effector function, similarly to what was previously reported for *Rag^−/−^* mice (30).

Expression of RAG may initiate prior to T and B cell differentiation, as indicated by the presence of incomplete rearrangements at the immunoglobulin heavy chain or T cell receptor loci in a minority of murine and human NK cells (29, 50). It has been suggested that introduction of DNA double strand breaks in lymphoid progenitor cells would facilitate selection of cells with more efficient DNA repair machinery (30). In the absence of this mechanism, NK cells from Rag-deficient animals and humans would have reduced cellular fitness. Our data indicate that indeed patients with mutations in *RAG* or in NHEJ genes share similar abnormalities of NK cell phenotype and function. While it is not known whether the immunoglobulin and T cell receptor loci represent the only sites in the genome that are targeted by RAG during early stages of lymphoid development, it is interesting to note that selective loss of CD56^dim^ cells, with relative preservation of the CD56^bright^ NK cell compartment, has been reported in patients with mutations of *MCM4* and *POLE2* genes, all of which control DNA replication (51–53).

In conclusion, we have identified for the first time abnormalities of NK cell phenotype and function in patients with mutations in *RAG* and in genes involved in NHEJ. These abnormalities are more pronounced in patients with severe mutations associated with a more severe clinical and immunological phenotype, and are not secondary to T and B cell lymphopenia. Demonstration of enhanced degranulation activity and higher amount of perforin in NK cells from patients with SCID due to RAG/NHEJ defects suggests that inclusion of serotherapy targeting NK cells in the HSCT conditioning regimen may help reduce the risk of graft rejection that has been reported in these diseases.

## Ethics Statement

The study was approved by the Institutional Review Boards of Boston Children’s Hospital, the National Institute of Allergy and Infectious Diseases (protocols 93-I-0119 and 16-I-N139), and of all other referring centers. Blood samples were obtained upon written informed consent of the subject or, in the case of minors, of their parents or legal guardians.

## Author Contributions

JM, EM, AM, SP, and LN designed the study, interpreted the data, and wrote the manuscript; KD, GT, EC, OP, PM, SG, and DM performed experiments, acquired and analyzed the data; WA-H, CC, MC, JB, CB, DB, SB, TC, JC, VD-C, LOdB, MTdlM, GM, AF, RG, RKG, AH, SH, C-HH, MK, AlKi, BK, AnKl, TK, BL, VL, MiMa, IM, MeMo, BN, S-YP, NP, AP, SP, IR, JS, RS, TT, Y-JK, JW, AG, and SK contributed patient samples and clinical and immunological data; all authors have revised the work for its intellectual content, have approved its final version and have agreed to be accountable for all aspects related to the accuracy and integrity of the work.

## Conflict of Interest Statement

The authors declare that the research was conducted in the absence of any commercial of financial relationship that could be construed as a potential conflict of interest.
