# Corrigendum: Natural Killer Cells from Patients with Recombinase-Activating Gene and Non-Homologous End Joining Gene Defects Comprise a Higher Frequency of CD56^bright^ NKG2A^+++^ Cells, and Yet Display Increased Degranulation and Higher Perforin Content

**DOI:** 10.3389/fimmu.2017.01244

**Published:** 2017-10-10

**Authors:** Kerry Dobbs, Giovanna Tabellini, Enrica Calzoni, Ornella Patrizi, Paula Martinez, Silvia Clara Giliani, Daniele Moratto, Waleed Al-Herz, Caterina Cancrini, Morton Cowan, Jacob Bleesing, Claire Booth, David Buchbinder, Siobhan O. Burns, Talal A. Chatila, Janet Chou, Vanessa Daza-Cajigal, Lisa M. Ott de Bruin, Maite Teresa de la Morena, Gigliola Di Matteo, Andrea Finocchi, Raif Geha, Rakesh K. Goyal, Anthony Hayward, Steven Holland, Chiung-Hui Huang, Maria G. Kanariou, Alejandra King, Blanka Kaplan, Anastasiya Kleva, Taco W. Kuijpers, Bee Wah Lee, Vassilios Lougaris, Michel Massaad, Isabelle Meyts, Megan Morsheimer, Benedicte Neven, Sung-Yun Pai, Nima Parvaneh, Alessandro Plebani, Susan Prockop, Ismail Reisli, Jian Yi Soh, Raz Somech, Troy R. Torgerson, Yae-Jean Kim, Jolan E. Walter, Andrew R. Gennery, Sevgi Keles, John P. Manis, Emanuela Marcenaro, Alessandro Moretta, Silvia Parolini, Luigi D. Notarangelo

**Affiliations:** ^1^Laboratory of Host Defenses, Division of Intramural Research, National Institute of Allergy and Infectious Diseases, National Institutes of Health, Bethesda, MD, United States; ^2^Department of Molecular and Translational Medicine, University of Brescia, Brescia, Italy; ^3^“A. Nocivelli Institute for Molecular Medicine”, Pediatric Clinic, University of Brescia, Azienda Socio Sanitaria Territoriale degli Spedali Civili di Brescia, Brescia, Italy; ^4^Hospital de Niños Ricardo Gutiérrez, Buenos Aires, Argentina; ^5^Department of Pediatrics, Faculty of Medicine, Kuwait University, Kuwait City, Kuwait; ^6^DPUO, Division of Immuno-Infectivology, University Department of Pediatrics, Bambino Gesù Children’s Hospital, Rome, Italy; ^7^School of Medicine, University of Tor Vergata, Rome, Italy; ^8^Pediatric Allergy Immunology and Blood and Marrow Transplant Division, University of California, San Francisco, Benioff Children’s Hospital, San Francisco, CA, United States; ^9^Division of Hematology/Oncology, Cincinnati Children’s Hospital Medical Center, Cincinnati, OH, United States; ^10^Institute for Immunity and Transplantation, University College London, London, United Kingdom; ^11^Division of Pediatric Hematology, Children’s Hospital Orange County, University of California Irvine, Orange County, CA, United States; ^12^Department of Immunology, Royal Free London NHS Foundation Trust, London, United Kingdom; ^13^Division of Immunology, Boston Children’s Hospital, Boston, MA, United States; ^14^Division of Allergy and Immunology, Southwestern Medical Center, University of Texas, Dallas, TX, United States; ^15^Division of Hematology/Oncology/BMT, Children’s Mercy Hospital & Clinics, Kansas City, MO, United States; ^16^Department of Pediatrics, Brown University, Providence, RI, United States; ^17^Laboratory of Clinical Infectious Diseases, National Institute of Allergy and Infectious Diseases, National Institutes of Health, Bethesda, MD, United States; ^18^Department of Paediatrics, National University Hospital, Singapore, Singapore; ^19^Department of Immunology-Histocompatibility, “Aghia Sophia” Children’s Hospital, Athens, Greece; ^20^Division of Pediatric Immunology, Hospital Luis Calvo Mackenna, Santiago, Chile; ^21^Department of Pediatrics, Division of Allergy and Immunology, Hofstra Northwell School of Medicine, Hofstra University, Great Neck, NY, United States; ^22^Department of Pediatric Hematology, Immunology and Infectious Diseases, Emma Children’s Hospital, Academic Medical Center (AMC), University of Amsterdam, Amsterdam, Netherlands; ^23^Department of Experimental and Clinical Sciences, University of Brescia, Brescia, Italy; ^24^Department of Pediatrics, University Hospitals Leuven, Leuven, Belgium; ^25^Transplantation Branch, Division of Allergy, Immunology and Transplantation, National Institute of Allergy and Infectious Diseases, National Institutes of Health, Rockville, MD, United States; ^26^Pediatric Hematology-Immunology Department, Hospital Necker-Enfants Malades, Institute Imagine, AP-HP, Paris Descartes University, Sorbonne-Paris-Cité, Paris, France; ^27^Division of Hematology-Oncology, Boston Children’s Hospital, Boston, MA, United States; ^28^Division of Allergy and Clinical Immunology, Department of Pediatrics, Tehran University of Medical Sciences, Tehran, Iran; ^29^Research Center for Immunodeficiencies, Children’s Medical Center, Tehran, Iran; ^30^Bone Marrow Transplant Service, Department of Pediatrics, Memorial Sloan Kettering Cancer Center, New York, NY, United States; ^31^Division of Pediatric Immunology and Allergy, Meram Medical Faculty, Necmettin Erbakan University, Konya, Turkey; ^32^Pediatric Immunology Unit, The Edmond and Lily Safra Children’s Hospital, Sheba Medical Center, Tel Hashomer, Sackler Faculty of Medicine, Tel Aviv University, Tel Aviv, Israel; ^33^Department of Pediatrics and Immunology, Seattle Children’s Hospital, University of Washington, Seattle, WA, United States; ^34^Division of Infectious Diseases and Immunodeficiency, Department of Pediatrics, Samsung Medical Center, School of Medicine, Sungkyunkwan University, Seoul, South Korea; ^35^Division of Pediatric Allergy/Immunology, University of South Florida at Johns Hopkins All Children’s Hospital, St. Petersburg, FL, United States; ^36^Department of Paediatric Immunology, Great North Children’s Hospital, Newcastle Upon Tyne, United Kingdom; ^37^Institute of Cellular Medicine, Newcastle University, Newcastle Upon Tyne, United Kingdom; ^38^Department of Laboratory Medicine, Boston Children’s Hospital, Boston, MA, United States; ^39^Molecular Immunology Laboratories, Department of Experimental Medicine, University of Genoa, Genoa, Italy

**Keywords:** natural killer cells, recombinase-activating genes, non-homologous end joining, immunodeficiency, CD56, interferon-γ, degranulation

There was a mistake in the authorship. The name of Nima Parvaneh was unintentionally omitted. Dr. Parvaneh has contributed biological specimens and clinical and immunological data from patient P66 included in the manuscript, and as such he should be included in the authorship. The authors apologize for the mistake. This error does not change the scientific conclusions of the article in any way.

With the inclusion of Dr. Parvaneh’s name in the authorship, the paragraph of Author Contributions should also be corrected as follows:

JM, EM, AM, SP, and LN designed the study, interpreted the data, and wrote the manuscript; KD, GT, EC, OP, PM, SG, and DM performed experiments, acquired and analyzed the data; WA-H, CC, MC, JB, CB, DB, SB, TC, JC, VD-C, LOdB, MTdlM, GM, AF, RG, RKG, AH, SH, C-HH, MK, AlKi, BK, AnKl, TK, BL, VL, MiMa, IM, MeMo, BN, S-YP, NP, AP, SP, IR, JS, RS, TT, Y-JK, JW, AG, and SK contributed patient samples and clinical and immunological data; all authors have revised the work for its intellectual content, have approved its final version and have agreed to be accountable for all aspects related to the accuracy and integrity of the work.

This correction does not change the scientific conclusions of the article in any way.

Author apologizes for these errors and thank you for your consideration.

The original has been updated.

## Conflict of Interest Statement

The authors declare that the research was conducted in the absence of any commercial or financial relationships that could be construed as a potential conflict of interest.

